# Functional Activated Biocarbons Based on Biomass Waste for CO_2_ Capture and Heavy Metal Sorption

**DOI:** 10.1021/acsomega.3c07120

**Published:** 2023-12-06

**Authors:** Jarosław Serafin, Kanagat Kishibayev, Rustam Tokpayev, Tamina Khavaza, Azhar Atchabarova, Zair Ibraimov, Mikhail Nauryzbayev, Joanna Sreńscek Nazzal, Liliana Giraldo, Juan Carlos Moreno-Piraján

**Affiliations:** † Institute of Energy Technologies, Department of Chemical Engineering and Barcelona Research Center in Multiscale Science and Engineering, Universitat Politècnica de Catalunya, Eduard Maristany 16, EEBE, Barcelona 08019, Spain; ‡ Center of Physical-Chemical Methods of Research and Analysis, Al Farabi Kazakh National University, 96 A, Tole bi Street, Almaty 050012, Kazakhstan; § Faculty of Chemical Technology and Engineering, Department of Catalytic and Sorbent Materials Engineering, West Pomeranian University of Technology in Szczecin, Piastów Ave. 42, Szczecin 71-065, Poland; ∥ Facultad de Ciencias, Departamento de Quimica, Grupo de Calorimetria Universidad Nacional de Colombia, Sede Bogota 111321, Colombia; ⊥ Facultad de Ciencias, Departamento de Quimica, Grupo de Investigación de Sólidos Porosos y Calorimetría, Universidad de los Andes, Bogotá 111711, Colombia

## Abstract

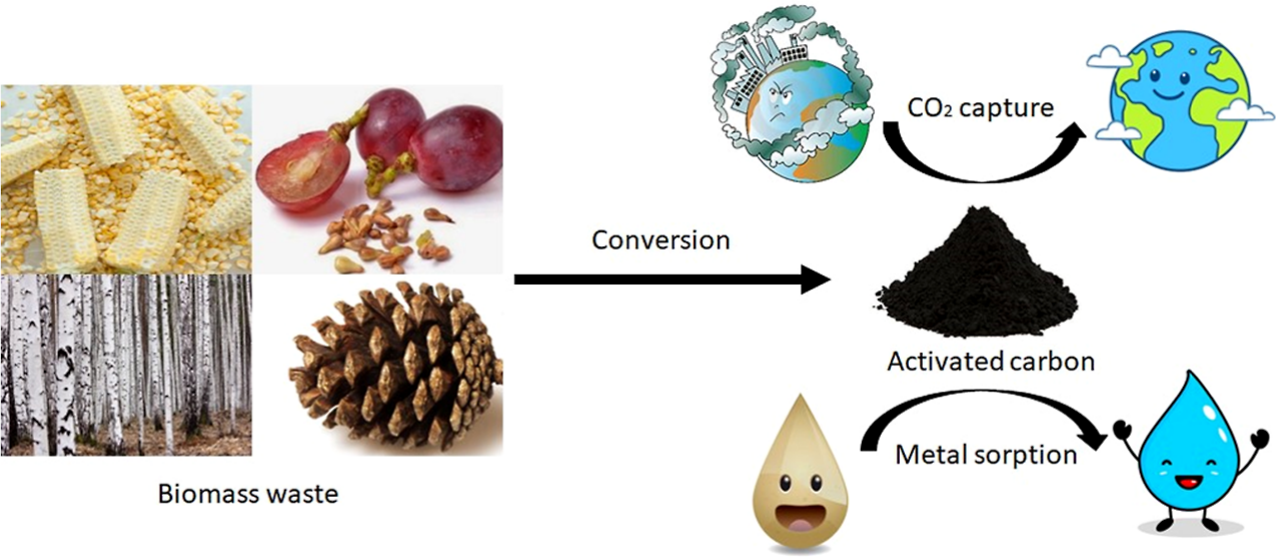

Inexpensive porous activated biocarbons were prepared from biomass and agriculture waste following the method of thermal and hydrothermal carbonization and activation with superheated water vapor. The activated biocarbons were characterized by nitrogen adsorption–desorption at 77 K, SEM, XRD, Raman spectrometry, FTIR spectroscopy, determination of particle size, and elemental composition by XRF. The specific surface area was in the range of 240–709 m^2^/g, and the total pore volume was from 0.12 to 0.43 cm^3^/g. The percentage of microporosity in activated biocarbons was 89–92%. These activated biocarbons have been used for CO_2_ and heavy metal sorption. Activated biocarbons based on pine cones and birch prepared by thermal carbonization and activation with superheated water vapor had the highest ability to capture CO_2_ and amounted to 6.43 and 6.00 mmol/g at 273 K, as well as 4.57 and 4.22 mmol/g at 298 K, respectively. The best activated biocarbon was characterized by unchanged stability after 30 adsorption and desorption cycles. It was proved that the adsorption of CO_2_ depends on narrow micropores (<1 nm). Activated biocarbons have also been analyzed as effective adsorbents for removing Cu^2+^, Zn^2+^, Fe^2+^, Ni^2+^, Co^2+^, and Pb^2+^ ions from aqueous solutions. Activated biocarbons are effective adsorbents for the removal of lead and zinc ions from aqueous solutions.

## 1 Introduction

In 2010–2011, much research focused on biocarbons and their potential applications in environmental purification. Early studies have shown that biocarbons have the potential as an ecological sorbent for removing various pollutants from soil, gas–air, and water systems.^1^ Furthermore, due to the relatively low cost and abundance of raw materials, including forestry and agricultural waste, biocarbons have become an alternative for cleaning various environmental pollutants, organic pollutants, including heavy metals, etc.^2,3^

However, the effectiveness of biocarbons in the sorption of CO_2_ and heavy metals from the environment varies significantly for different forms of biocarbons. It can be controlled by a few elements, such as type of raw materials, method of production, and/or treatment conditions. Generally, biocarbons obtained directly from plant biomass waste without preliminary or subsequent processing have a relatively low sorption capacity for CO_2_ and heavy metals. Thus, different activation and/or modification methods, such as surface oxidation and grafting of functional groups, are used to improve their effectiveness in restoring the environment.^4,5^ These activations/modifications can increase the number of surface sorption centers, in particular, by adding surface functional groups, which can be the dominant factor of the adsorption of CO_2_ and heavy metal ions by biocarbons.^6^

Carbon dioxide O_2_ concentrations will rise from preindustrial levels of 280–384 ppm in 2007, and by 2050, they are expected to reach 550 ppm, even though CO_2_ emissions will remain stable over the next 40 years. One of the CO_2_ concentrations in the atmosphere is global warming. In fact, global warming has increased significantly over the past 50 years.^7,8^

Currently, the attention of scientists is focused on the search for and creation of new, more efficient, and economical adsorbents for capturing CO_2_. Zeolites, covalent organic framework, metal–organic frameworks, materials based on alkali metals, solid sorbents based on amines, and carbon-containing materials were mainly investigated as potential CO_2_ sorbents.^9−12^ Activated biocarbon is considered to be one of the encouraging solid adsorbents that can be used to capture CO_2_ due to its many advantages, such as low price, ease of regeneration, high ability to adsorb CO_2_ under ambient conditions, high specific surface area, required porosity, and low energy consumption.^13,14^ Many studies are devoted to the development of carbon-containing adsorbents received based on agricultural biomass.^15−20^ The wide availability of agricultural byproducts makes them a good raw material to produce inexpensive activated carbons to capture carbon dioxide.

Heavy metals are a group of elements, that is, metals with an atomic density of more than 4 ± 1 g/cm^3^, for example, copper, zinc, mercury, cadmium, lead, tin, iron, manganese, chromium, cobalt, nickel, arsenic, and aluminum. The ions of these metals are the most common toxic mineral pollutants in soils and water environments.^21,22^

Reasonable use of water resources seems to be one of the world’s environmental problems, and it is mainly solved by removing wastewater from human activities in various industries. Controlling heavy metal content is one of the most harmful and toxic components of wastewater in the biological world.^23,24^

There are two main sources of heavy metals in wastewater: anthropogenic and natural. The first includes volcanic activity, soil erosion, and weathering of minerals. On the contrary, the latter includes fuel combustion, mineral processing, street runoff, landfills, agricultural activities, and industrial activities. Due to the stability, high solubility, and migration activity of heavy metals in aquatic environments, untreated or insufficiently treated wastewater contaminated with metals, when released into water bodies, has a variety of effects on human health and the environment.^25^ Plants adsorb heavy metals, entering the body of animals and humans through food chains and negatively affecting their vital activity and health.^25−27^ The structure of the electron shells of the atoms of these pollutants determines their high reactivity, tendency to complex formation, and, as a result, high physiological and biochemical activity, which leads to a number of environmental and medical consequences. The negative effects of heavy metals on plants include reduced seed germination and lipid content due to cadmium, reduced enzyme activity and plant growth due to chromium, inhibition of photosynthesis by copper and mercury, reduced seed germination due to nickel, and reduced chlorophyll production and plant growth due to lead.^25^ Heavy metals cause serious health effects in humans and animals such as stunted growth and development, cancer, organ damage, damage to the nervous system, and, in extreme cases, death. Exposure to some heavy metals, such as mercury and lead, can also cause autoimmunity, in which a person’s immune system attacks its own cells. This can lead to diseases of joints such as rheumatoid arthritis and diseases of kidneys, circulatory system, and nervous system. At higher doses, heavy metals can cause irreversible brain damage.^26^ Therefore, it is mandatory to treat wastewater contaminated with heavy metals before discharging it into the environment in order to avoid such negative repercussions as getting into drinking water.

Heavy metals can be removed from aqueous media by different methods, such as chemical precipitation, ion exchange, membrane filtration, solvent extraction, coagulation, and electrochemical removal, etc. However, these methods have several disadvantages, such as incomplete removal, high energy consumption, presence of toxic sludge, sensitive operating conditions, low efficiency, and expensive disposal.^28^

Currently, adsorption is considered an effective and inexpensive method for removing harmful heavy metal ions from wastewater. This process is considered flexible in design and operation and allows us to obtain of high-quality treated effluents.^29^

Commercial activated biocarbon, a traditional and effective adsorbent, is used worldwide to remove all kinds of contaminants, but its use is limited due to high associated costs.^30^ Therefore, scientists are turning their interest in finding other inexpensive raw materials for producing alternative activated biocarbons. Agricultural waste/biomass products are generally renewable, inexpensive, nontoxic, and environmentally friendly carbonaceous materials.^31^ In this regard, in many literature sources, various plant biomass wastes are used, such as sugar cane waste,^32^ cotton,^33^ sunflower,^34^ pistachios,^35^ fruit waste,^36^ walnut shells,^37^ coconut shells,^38^ and watermelon peels,^39^ for the production of adsorbents used in the sorption of CO_2_ and heavy metals.

The production of activated biocarbons usually involves two stages: carbonization and physical or chemical activation of the carbonized substances produced. Physical activation includes the oxidation and gasification of char at high temperatures. In the process of chemical activation, carbonization and activation are performed in one stage by thermal decomposition of raw materials impregnated with some chemicals, such as ZnCl_2_, NaOH, KOH, K_2_CO_3_, and H_3_PO_4_.^40−44^ When activated, the textural characteristics of the materials may also change. It was shown that the sorption capacity of materials increases as a result of the activation process.^45^ In addition, physical activation allows better control over the process of creating the desired microporous structure. Activation with superheated water vapor (SWVA) showed the best possibilities for obtaining activated biocarbons with a high specific surface area and a good pore ratio.^46^

In this article, we propose a simple method for converting waste biomass into activated biocarbons, which is a key problem associated with their disposal. Activated biocarbons were prepared by thermal carbonization (TC) and hydrothermal carbonization (HTC), followed by activation with SWVA. Then, we studied the sorption of CO_2_ and heavy metals on the obtained activated biocarbons. Activation by SWVA is an economical method. This provides better activation and increases the extent of expansion of a narrow network of micropores at higher temperatures. To study the sorption properties of the obtained activated biocarbons with respect to CO_2_ and heavy metals under various conditions, sorption experiments were carried out. The purpose of this study was to synthesize and determine the main physical and chemical characteristics of activated biocarbons based on vegetable raw material waste obtained by thermal, HTC, and activation with SWVA, as well as to demonstrate the adsorption efficiency of activated biocarbons obtained from waste plant materials by activation with SWVA, to capture CO_2_ from the gas–air environment, and remove heavy metals from water and wastewater.

Almaty is characterized by a rather difficult environmental situation due to its location in a foothill basin, which leads to strong air pollution. One of the main sources of CO_2_ emissions in Almaty is CHP-1 and CHP-2 located in the city due to the burning of fossil fuels as well as a large number of vehicles that also release carbon dioxide into the environment.

Carbon dioxide is one of the most important greenhouse gases causing the effects of global warming and climate change and also causes breathing difficulties at elevated concentrations. In this regard, the capture of CO_2_ from gas–air media is an actual task at the present time. Metallurgical production is also developed in the Republic of Kazakhstan, which produces copper, zinc, lead, etc.; in this regard, there is a need to purify industrial wastewater from heavy metals for their further use in production cycles and to reduce environmental pollution. Heavy metals are toxic substances that, at high concentrations, have a negative impact on the environment and human health. In this regard, the search for cost-effective ways to clean gas–air media from carbon dioxide, as well as wastewater from heavy metal ions, is an important and actual task at the present time. One such method is sorption on activated biocarbons. Activated biocarbons obtained in this work are cost-effective, as they are prepared from plant wastes, which are renewable raw materials and are available in sufficient quantities in the Republic of Kazakhstan. Currently, activated biocarbons are not widely used in Kazakhstan for cleaning gas–air environments from carbon dioxide and wastewater from heavy metal ions.

## 2 Materials and Methods

In this study, corn cobs (CC), grape seeds (GS), and birch and pine cones (PC) were used as raw materials to obtain activated biocarbons, which were prepared by thermal carbonization and activation with SWVA. Activated biocarbons based on PC have also been produced by HTC and activation with SWVA. The choice of these plant materials as a raw material to produce activated biocarbons is due to the fact that agriculture is developed in Kazakhstan; the waste of these plant materials is generated in large quantities, and this raw material is renewable and not expensive. CC and GS were collected in the city of Zharkent, Zhetysu region (Kazakhstan). PC and birches were collected in the mountainous areas of Almaty (Kazakhstan).

### 2.1 Preparation of Activated Biocarbons Based on Plant Raw Materials’ Waste by Thermal Carbonization and Activation with SWVA

To prepare activated biocarbons from biomass and agriculture raw material waste, they were preliminarily crushed into particles (size of 2–3 mm) using a rotary knife mill, RM-120 (Vibrotechnik, Russia). A horizontal reactor was used for the carbonization process at 700 °C/1 h using argon with a flow of 10 mL/min like an inert gas. The mass of the raw material was approximately 200 g.

The prepared carbonized materials were activated with SWVA to improve their surface area and porosity structure. Activation was carried out in a vertical quartz glass reactor; the weight of the carbonized material was approximately 75 g. The activation process was performed at 700 °C with an exposure of 1 h at this temperature. Therefore, of the activation of biomass and agriculture waste-carbonized materials, the specific surface area and porosity of the samples significantly increase.^47^

The finally prepared activated biocarbons are called CC (TC + SWVA)—from CC, GS (TC + SWVA)—from GS, birch (TC + SWVA)—from birch trunk, and PC (TC + SWVA)—from PC.

### 2.2 Preparation of Activated Biocarbons Based on Plant Raw Material Waste by HTC and Activation with SWVA

Activated biocarbon from PC was also prepared by HTC. The raw materials were preliminarily crushed to a working particle of 2–3 mm in a rotary knife mill, RM-120 (Vibrotechnik, Russia). Further, the samples were washed and dried at 100–105 °C for 2 h and then subjected for HTC. HTC was carried out in a sealed steel autoclave cup at a temperature of 220 °C in an aqueous medium, a pressure of 2 MPa occurs as a result of the formation of water vapor pressure over the liquid and the carbonized material when heated for 24 h, and the ratio S/L 1:2 is the ratio of carbonized material to water, which are loaded into an autoclave cup. The process of HTC is carried out as follows: heating the autoclave up to a temperature of 220 °C for 1.5–2 h, then the temperature (220 °C) and hence the pressure (2 MPa) were supported constantly for 24 h, with further cooling of the autoclave cup in the open air, and the pressure and temperature were reduced to normal conditions for 6–8 h. After the reactor was cooled to room temperature, the pressure was released, and the wet mass was filtered and dried at 100–105 °C for 2 h.

The prepared carbonized materials were also activated with SWVA at 700 °C for 1 h in the reactor described in Section 2.1.^47^

The obtained activated biocarbon was named PC (HTC + SWVA)—was from PC.

In this work, the activation of carbonized wastes of plant raw materials was carried out at 700 °C because at this temperature, a high yield of activated biocarbon is obtained. When 75 g of carbonized plant material is activated with SWVA, approximately 37 g of activated biocarbon is formed, and when activated at higher temperatures, the yield of activated biocarbon decreases. Also, upon activation at 700 °C, a developed microporous surface structure, a high specific surface area, and a low ash content are formed. At 700 °C, we obtain activated biocarbons with the required specific surface area and porosity, which are necessary for good CO_2_ capture and heavy metal sorption. After all, one of the advantages of physical activation is the independent regulation of the surface area and porosity of the obtained activated biocarbons by varying the activation temperature for the appropriate adsorption of various pollutants.

### 2.3 Characterization of Activated Biocarbons

Textural characteristics of activated biocarbons were obtained using nitrogen sorption at −196 °C. Experiments were carried out using a Quadrasorb evo device for volumetric gas adsorption/desorption. Previously, the samples were degassed at 250 °C and a pressure of 1 × 10^–6^ bar during 12 h. Specific surface area (*S*_BET_) was examined using the (Brunauer–Emmett–Teller) (BET) equation. The total pore volume (*V*_total_) was calculated based on the amount of nitrogen adsorbed at the highest relative pressure (1 bar). The pore size distribution (PSD) below 1.4 nm was calculated using CO_2_ adsorption isotherms, applying a nonlocalized density functional theory model.

A scanning electron microscope (UHR FE-SEM Hitachi SU8020) equipped with secondary electron detectors (SE) and a four-quadrant photodiode detector of backscattered electrons (PD-BSE) were used to obtain microphotographs of activated biocarbons. Transmission electron microscopy (TEM) (JEOL 2100 with a high tension of 200 kV and *a* point resolution of 0.24 nm) was performed.

X-ray diffractograms of activated biocarbons were obtained using an analytical X-ray “PANalytical” device, a Philips analytical diffractometer with Cu Ka radiation (λ—0.1542 nm). The angular velocity was 0.033 s with a step of 0.02. Dried activated biocarbon samples were ground with a pestle in an agate mortar. X’Pert High-Store diffraction software was used for analysis.

To determine the quality of the prepared activated biocarbon, Raman spectroscopy was used. The measurement was performed on a Renishaw InVia Raman Microscope equipped with a CCD detector and a laser with a laser length of 785 nm.

Fourier spectroscopy from The Digilab Division of Bio-Rad (Cambridge, Massachusetts, USA), model FTS 175 C, was used to determine activated biocarbon spectra in the measurement range of 4000–500 cm^–1^.

Mastersizer 3000 granulometer (Malvern Instruments) equipment was used to estimate the particle size distribution of activated biocarbons. The samples were dispersed in powders in water under mechanical stirring of the suspension.

For elemental analysis in activated biocarbons, it was carried out with elemental analyzers (LECO Corporation).

The X-ray fluorescence energy dispersion spectrophotometer (EDXRF) of Epsilon 3 type from PANalytical B was used to determine the content of elements from sodium (Na, Z = 11) to uranium (U, Z = 92) in activated biocarbons.

The ash content of activated biocarbons was estimated in accordance with the standard described in the article.^47^

The ash content in the activated biocarbon samples was estimated using eq 1
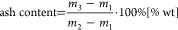
1where *m*_1_ is the weight of the calcined container, [g]. *m*_2_ is the weight of a container with an amount of activated biocarbon, and [g]. *m*_3_ is the weight of the container with ash, [g].

### 2.4 Evaluation of CO_2_ Adsorption

To assess the CO_2_ adsorption capacities of hydrochars and activated carbons, we used a gas porosimetry system to determine the CO_2_ uptake as a function of the pressure change. Higher adsorption uptake was observed at a lower temperature (273 K), which can be attributed to the exothermic nature of the CO_2_ adsorption process.

The temperature for adsorption was achieved by using a Dewar, having a circulating jacket, and connected to a thermostatic bath. Before each adsorption experiment, the carbon sample was outgassed at 200 °C for 12 h under vacuum. The CO_2_ adsorption capacity was expressed in mmol CO_2_/g adsorbent.

### 2.5 Study of pH and Determination of Isotherms of Heavy Metal Adsorption from Aqueous Solution

The adsorption experiments from aqueous solution were performed using pyrex glass flasks specially designed for this research; they were conical in shape in order to separate the possible precipitated material during the magnetic stirring process at 100 rpm and to achieve a good dispersion. These containers were placed in a water thermostat to control the temperature 25 ± 0.5 °C for 8 days in order to ensure that each solution reached complete equilibrium between adsorbate–adsorbent [initial metal ion concentration (*C*_o_) = 10–100 mg/L and adsorbent dose = 0.01 g/50 mL. The pH value was chosen so that metallic species are present in their divalent form (pH = 5.0), Pb^2+^, Zn^2+^, Cu^2+^, Fe^2+^, Ni^2+^, and Co^2+^]. During the experiments, the pH value was adjusted by adding 0.1 mol/L HCl or NaOH solutions, and the mixture was stirred at 300 rpm. Then, the respective analyses were carried out at equilibrium in solution, and their concentrations were established taking into account the respective calibration curves for each ion, previously constructed. This solution is infused continuously for at least 8 days, which is considered the maximum contact time for achieving a proper balance between the size of the sorbent and particles (according to previous studies on many other metals). Blank samples were also processed in the absence of sorbent to check sorption in experimental equipment (packers, filters, etc.) and in the absence of precipitation phenomena. Finally, the sample was collected and filtered with a 1.2 μm filter membrane with a pore size. The atomic emission spectrometry (ContrAA 800-High-End AAS Spectrometer, Analytik Jena, United States) measured the content of metal ions in the filter. To calculate the amount of each ion at equilibrium, the mass balance equation was used. Thus, the adsorption capacity *q* (mg ions/g) was obtained from
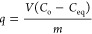
2where *V* is the volume of the solution (L), *m* is the amount of adsorbent (g), *C*_o_ and *C*_eq_ are the initial and equilibrium concentrations in the solution (ion, mg/L), respectively, and *q* is the equilibrium concentration of metal ions onto samples (mg/g).

Blank solution is treated in the same way without the sorbent, and the registered concentration at the end of each operation is considered an initial concentration.

The adsorption percentage (% removal) of metal ions from aqueous solution is computed as follows
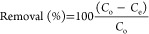


## 3 Results and Discussion

### 3.1 Physicochemical Characteristics of Activated Biocarbons Obtained from Biomass and Agriculture Raw Material Waste

**Figure 1:**
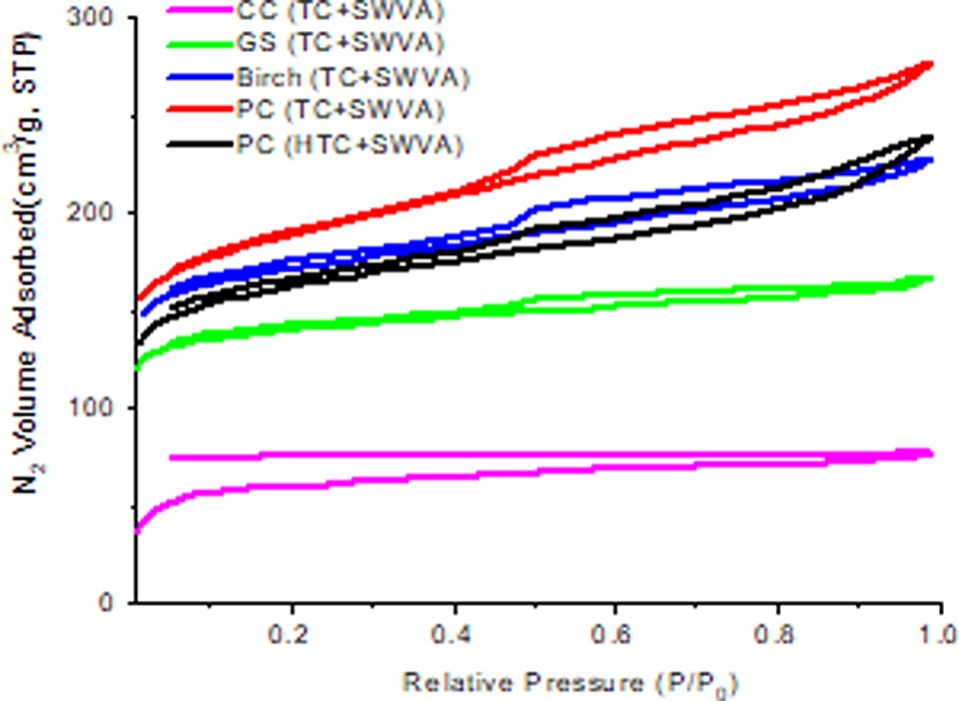
Isotherms of adsorption and desorption of nitrogen at 77 K by activated biocarbons based on plant wastes.

Textural characteristic analysis provides valuable information about the surface area and pore structure of activated carbons, which is crucial for evaluating their adsorption capacity. Figure 1 shows nitrogen adsorption–desorption isotherms obtained by activated biocarbons from plant waste at a temperature of 77 K.

All activated biocarbons based on biomass and agriculture wastes obtained by thermal, HTC, and activation with SWVA showed two types of isotherms, types I and IV (Figure 1). CC (TC + SWVA)-activated carbon represents type I and the rest one its typical IV type of isotherm adsorption. Nitrogen adsorption is significant at low pressures, the isotherm bend is more open, and a plateau forms at higher relative pressures. According to the classification of the International Union of Pure and Applied Chemistry (IUPAC), these isotherms belong to type I, which is microporous material with a relatively large outer surface area.^48^ The specific surface area of activated biocarbons from plant biomass waste according to BET ranges from 240 to 709 m^2^/g (see Table 1). Visible hysteresis loop indicates that prepared biocarbons have micropores and also some mesopores. The hysteresis loop subsists in a high relative region (*P*/*P*_0_ > 0.41). This isotherm shows an H4-type hysteresis loop. For type H4, the hysteresis loop has lamellar particles forming slit-like pores.^49^

**Table 1:** Textural Characteristics of Activated Biocarbons Based on Plant Raw Material Waste

activated biocarbons	*S*_BET_ [m^2^/g]	*V*_total_ [cm^3^/g]	*V*_mN_2__ [cm^3^/g]	*V*_mCO_2__ [cm^3^/g]	percentage of micropores
CC (TC + SWVA)	240	0.12	0.11	0.16	92
GS (TC + SWVA)	549	0.26	0.23	0.19	89
Birch (TC + SWVA)	662	0.35	0.32	0.23	90
PC (TC + SWVA)	709	0.43	0.39	0.21	90
PC (HTC + SWVA)	617	0.37	0.34	0.19	90

**Figure 2:**
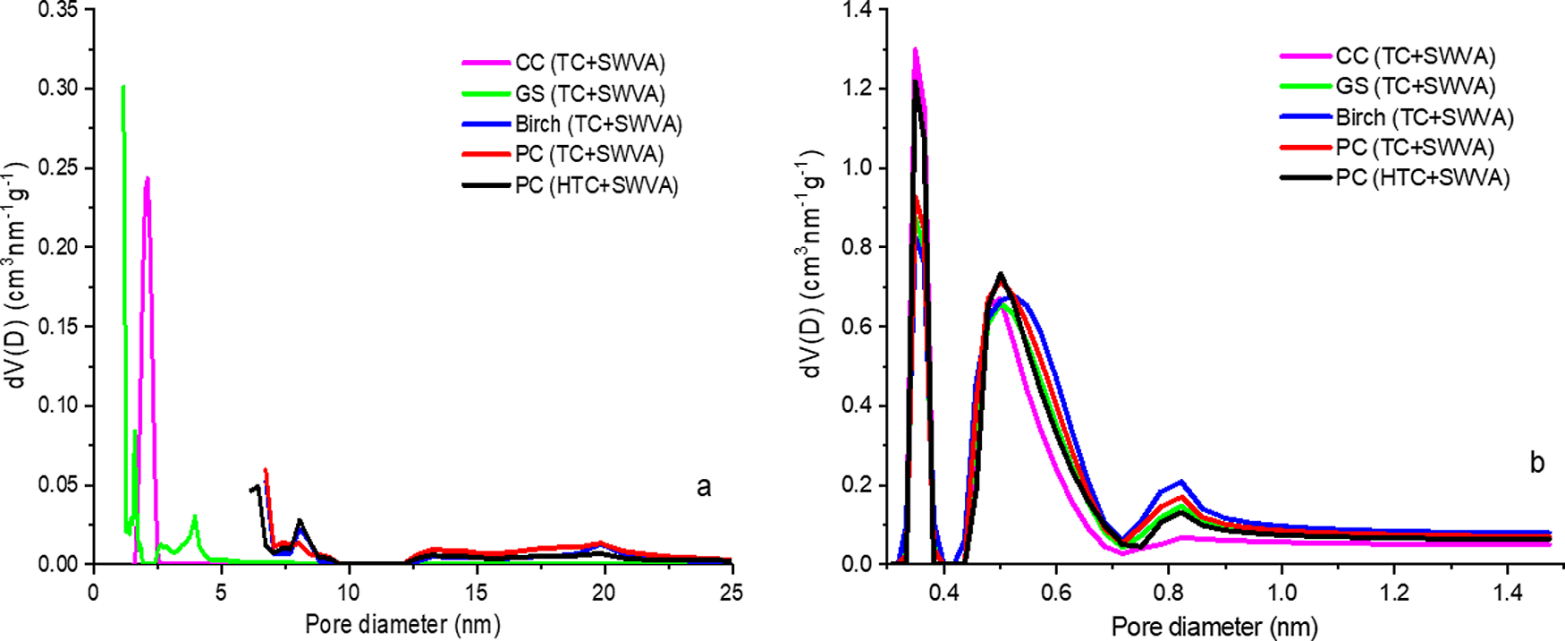
PSD of activated biocarbons based on plant material wastes obtained from (a) N_2_ adsorption at 77 K and (b) CO_2_ adsorption at 273 K.

Figure 2a presents the PSD determined by the DFT method by measuring the N_2_ adsorption at 77 K. Figure 2b shows the distribution of pores in the range 0.3–1.4 nm, as determined by the method of DFT by adsorption of CO_2_ at 273 K. The pore distribution and PSD of the obtained activated biocarbons (Figure 2a,b) were narrowly distributed in the range of micromesopores.

The PSD is estimated from the analysis of nitrogen adsorption isotherms measured at 77 K. At low temperatures, the distribution of nitrogen molecules into small pores is very slow, and small pores less than 1 nm cannot be evaluated. This problem can be solved by using CO_2_ adsorption at 273 K. Therefore, the low relative pressure measurement required to analyze small micropores can be obtained at a moderate absolute pressure. At higher temperatures and absolute pressures, the CO_2_ molecules are easier to penetrate than N_2_ molecules at 77 K, although the critical sizes of both gases are the same. Nitrogen adsorption can be used to estimate the diameter of the pores with about 1.1–100 nm. CO_2_ vaporization provides information about the diameter of the pores at 0.3–1.1 nm. The assessment of small pores (less than 1.2 nm) with CO_2_ adsorption at 273 K is a common method described and used by many authors.^50,51^

According to the results of the distribution of pores by size determined by nitrogen adsorption at 77 K (Figure 2a), it is found that activated biocarbon GS (TC + SWVA) micropores with a size of 1.3–1.6 nm and mesopores with a size of 2.6–4 nm predominate. Activated biocarbon CC (TC + SWVA) (Figure 2a) mainly has micropores of 1.6 nm and mesopores of 2.4 nm, while other activated biocarbons birch (TC + SWVA), PC (TC + SWVA), and PC (HTC + SWVA) (Figure 2a) contained mesopores ranging in size from 6.4 to 20 nm. Pores are formed during the release of volatile substances, including the release of carbon-containing compounds during carbonization, as well as under the action of SWVA as an activator.^47^

Obtained activated biocarbons contained micropores in their structure with a large contribution of pores in the submicropore (less than 0.4 nm), ultramicropore (0.4–0.7 nm), and supermicropore (0.7–2 nm) ranges, which were determined using CO_2_ sorption at 273 K (Figure 2b).^52,53^ These narrow micropores are observed in all activated biocarbons, regardless of the method of carbonization and activation. An analysis of the PSD (Figure 2a,b) confirms the structural changes in the studied activated biocarbons. In the case of these samples, prepared using thermal and HTC and activation with SWVA as an activator, materials with a predominantly microporous structure and a well-developed specific surface area were obtained. This characteristic of activated biocarbons is particularly advantageous from an environmental point of view since it allows both small and medium molecules of various pollutants to be efficiently adsorbed in single-component or multicomponent systems.^47^

**Figure 3:**
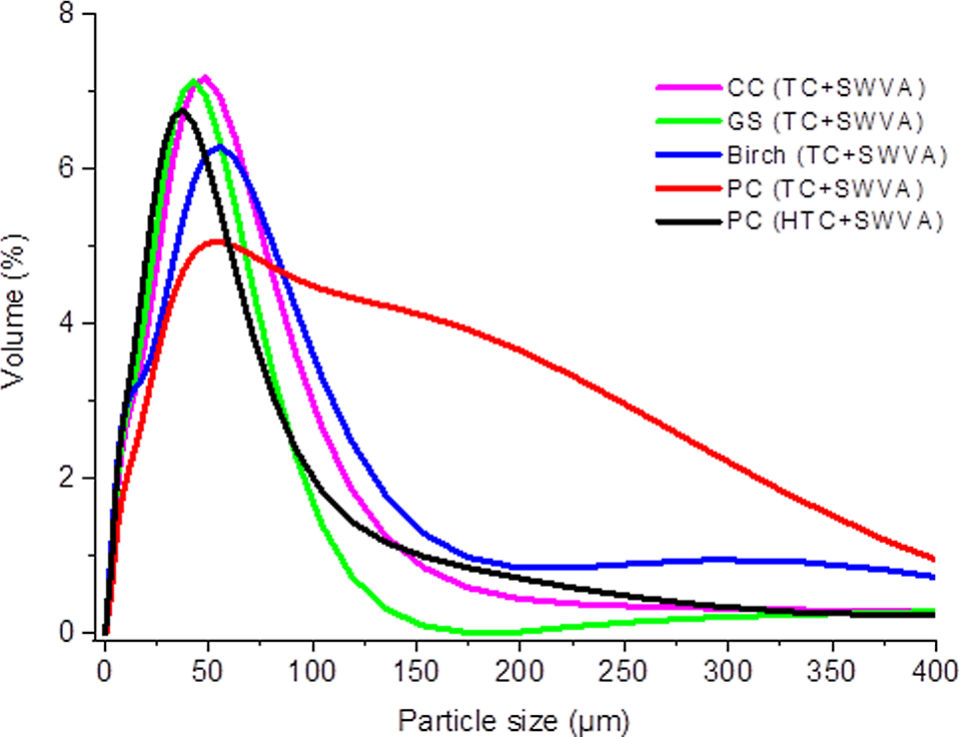
Particle size distribution of activated biocarbons based on the waste of plant materials.

Figure 3 shows the results of the particle size distribution of activated biocarbons based on waste of plant materials.

According to the particle size distribution (Figure 3), activated biocarbons based on the waste of plant materials obtained by thermal, HTC, and SWVA activation had a diameter of 20–60 μm, and more than 80% of the particles were smaller than 60 μm. Access to the specific surface area is better when the activated biocarbon particle size is smaller.^54^

Fine particles with high specific surface area and unique characteristics are very interesting for many applications. Controlling of their size, shape, coherence, and composition is necessary and important to ensure their specific commercial applications.^55^ Therefore, in recent years, there has been an increased interest in the production of specific adsorbent materials characterized by fine or ultrafine particles. As Raganati and Ammendola pointed out in 2021,^56^ these materials may well serve as a substrate for the production of sorbents with an unusual affinity for CO_2_ molecules^57^ and heavy metal ions. Their advantages include physicochemical properties and easy of modification.^58^

Table 1 shows the textural parameters of the activated biocarbons: the specific surface area (*S*_BET_), total pore volume (*V*_total_), micropore volume from N_2_ adsorption at 77 K (*V*_mN_2__), CO_2_ adsorption at 273 K (*V*_mCO_2__), and also percentage of microporosity of the obtained activated biocarbons.

From Table 1, it is visible that the obtained activated biocarbons from plant raw material waste have values of specific surface area and total pore volume in the range 240–709 m^2^/g and 0.12–0.43 cm^3^/g, respectively. The micropore volume examined by nitrogen adsorption ranged from 0.11 to 0.39 cm^3^/g, and the micropore volume found by CO_2_ adsorption ranged from 0.16 to 0.23 cm^3^/g. Activated biocarbons based on PC (TC + SWVA) (*S*_BET_ = 709 m^2^/g, *V*_total_ = 0.43 cm^3^/g, *V*_mN_2__ = 0.39 cm^3^/g, and *V*_mCO_2__ = 0.21 cm^3^/g) and birch (TC + SWVA) (*S*_BET_ = 662 m^2^/g, *V*_total_ = 0.35 cm^3^/g, *V*_mN_2__ = 0.32 cm^3^/g, and *V*_mCO_2__ = 0.23 cm^3^/g) have a high specific surface area, total pore volume, micropore volume determined by N_2_ adsorption, and micropore volume found by CO_2_ adsorption. The obtained results (Table 1) also show that activated biocarbon based on CC, GS, birch, and PC obtained by thermal and HTC and activation by SWVA has a highly microporous structure. The percentage of microporosity of all obtained activated biocarbons was in the range of 89–92%. It was found that thermal carbonization and SWVA activation of waste of plant material-specific surface area and total pore volume increase as the flow of argon and SWVA increases to 10 and 33 mL/min, respectively, and the temperature of carbonization and activation is 700 °C. In this connection, we assume that the difference in specific surface area and porosity of the obtained activated biocarbons is related to the nature and structure of the plant materials used. The obtained activated biocarbons prevail the volume of micropores up to 1.4 nm in size in terms of CO_2_, which is confirmed by the graphs of PSD received by CO_2_ adsorption (Figure 2b).^59^

**Figure 4:**
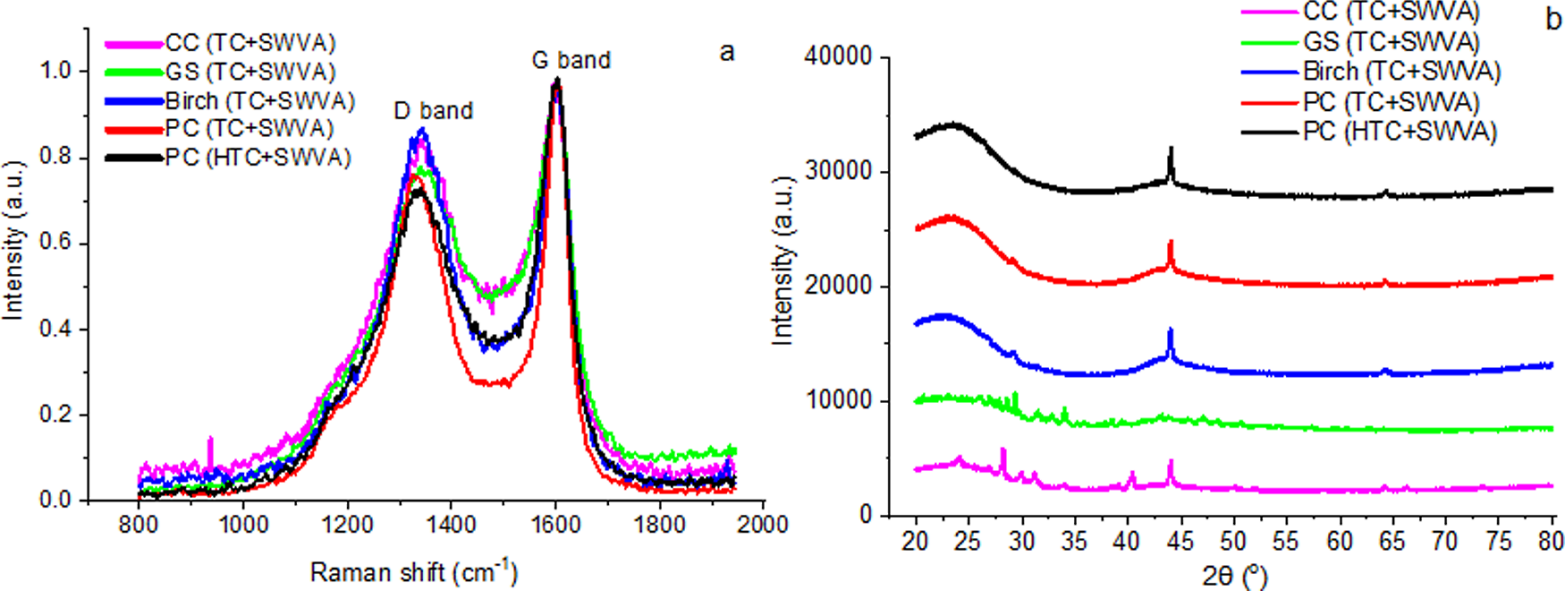
Characteristics of activated biocarbons using (a) Raman spectra and (b) X-ray diffractograms.

Figure 4 shows the characteristics of activated biocarbons obtained from plant waste by using Raman spectra (Figure 4a) and X-ray diffractograms (Figure 4b).

Raman spectroscopy is employed to investigate the vibrational and structural properties of activated carbons, providing essential data on their molecular arrangement and surface functional groups and aiding in the understanding of their reactivity and adsorption behavior. The Raman spectra of carbon (Figure 4a) are dominated by relatively sharp D and G bands. For each carbon material, the G band was present, and as the disorder in the turbostratic structure increased, another band, denoted as the D band, appeared. The investigated activated biocarbons prepared from biomass and agriculture waste have two characteristic bands in the frequency range of about 1600 cm^–1^ (the G band) and about 1305 cm^–1^ (the D band). The G band can be attributed to carbon–carbon vibrations in the plane of the graphite layers, and the D band presumably arises from an imperfect turbostratic structure. The intensity of the D band is proportional to the number of defects in the carbon structure.^60^ It was observed that the G band is more intense than the D band, which confirms the high carbon–carbon variation in the plane of the graphite layers and the turbostratic structure of the obtained activated biocarbons. Analysis of activated biocarbons by Raman spectroscopy confirmed the results of X-ray diffraction (XRD) studies (Figure 4b).

X-ray diffractograms are used to investigate the crystallinity and crystallographic structure of activated carbons, helping researchers assess their thermal stability and compatibility with specific applications. Figure 4b shows the X-ray diffractograms and their corresponding peaks in the range studied for the materials prepared in this investigation. It is highlighted from XRD that toward values of 2θ = ∼23°, the presence of a broad band that corresponds to the disordered layers that are characteristic of activated carbons, a material that is worth mentioning, is very amorphous as observed in this part of XRD. Toward 2θ = ∼44°, some sharp peaks of lower intensity are observed for all samples that correspond to traces of minerals in the starting lignocellulosic materials and to a slight organization that occurs in these materials during pyrolysis.^61^ When evaluating the Miller indices of the most relevant peaks, they show values of (002) and (101) for the broad band and these peaks. It is worth highlighting that during the preparation of an activated carbon, the decomposition products, in addition to general porosity and disorganization of the structure, may occur from a textural perspective in some parts of the porous material that have a certain organization, which is why it is possible with DRx calculate Miller indices. XRD also shows (Figure 4b) that the obtained activated biocarbons exhibit a homogeneous structural change. The peaks characterize the crystalline structure of the graphite sample.^47^ The α-Fe phase corresponds to the peaks at 2θ = ∼65° that were observed for activated biocarbons from birch and PC.

Other peaks are also visible in the XRD spectra, and we assume that there may be impurities of metal compounds left after carbonization and activation. Metallic impurities are observed in the XRD spectra of activated biocarbons based on CC and GS. In the activated biocarbons from corn cob and GS, we observed peaks at the following 2θ values of 24, 27, 28, 30°, 31, 34, and 40°, and we assume that this refers to magnetite (Fe_3_O_4_), iron(III) phosphate (FePO_4_), magnesium silicate, magnesium carbide (Mg_2_C_3_), Mg(OH)_2_, and MgO.^62,63^

In summary, we see a characteristic XRD of a disordered material such as activated carbons that allows the impurities of the starting materials to be recorded at average values of 2θ.

X-ray fluorescence energy analysis is used to quantify the elemental composition and impurity levels in activated carbons, contributing to a comprehensive assessment of their chemical composition and potential contaminants. In activated biocarbons based on CC and GS, other small peaks of impurities of various elements are observed, and we assume that these can be impurities of phosphorus, potassium, and calcium, whose content on the surface of activated biocarbon is confirmed by X-ray fluorescence analysis (Table 3). The obtained results found that the observed elements’ impurities are not completely removed after the carbonization and activation processes but are converted into various compounds due to interaction with activating agents at high temperature. The presence of Fe nanocrystals is explained by the reduction of Fe_3_O_4_ by amorphous carbon formed during carbon pyrolysis. It was found that the intensity of the 2θ peaks corresponding to Fe_3_O_4_ and α-Fe also increased with increasing Fe content.^64^

**Table 2:** Main Elemental Analysis of Activated Biocarbons

		concentration of elements [%]			
no	activated biocarbons	C	H	N	O
1	CC (TC + SWVA)	88.74	0.89	1.23	9.14
2	GS (TC + SWVA)	87.32	0.79	1.12	10.77
3	birch (TC + SWVA)	90.94	0.84	0.99	7.23
4	PC (TC + SWVA)	91.45	0.91	1.02	6.62
5	PC (HTC + SWVA)	89.59	0.87	1.17	8.37

**Table 3:** Elemental Composition by the XRF Method and Ash Content of Activated Biocarbons

		concentration of elements [%]						
no	activated biocarbons	Mg	Si	P	K	Ca	Fe	ash content [%]
1	CC (TC + SWVA)	0.11	0.78	0.16	0.12	0.09	0.13	11.15
2	GS (TC + SWVA)	0.99	0.10	0.26	0.45	0.17	0.11	0.62
3	Birch (TC + SWVA)	0.22	0.11	0.11	0.17	0.52	0.19	2.12
4	PC (TC + SWVA)	0.46	0.44	0.16	0.53	0.43	0.20	4.72
5	PC (HTC + SWVA)	0.43	0.26	0.12	0.11	0.27	0.47	2.72

Elemental analysis is crucial in determining the elemental composition of activated carbons, offering insights into potential impurities and the material’s overall chemical makeup. The activated biocarbons were analyzed to known elemental analysis, and the results are shown in Tables 2 and 3. For all carbon samples, high contents of C and lower contents of H and O were observed. This means that thermal carbonization in an inert atmosphere and physical activation involving SWVA led to accelerate the removal of H and O, and this resulted in an increased C. The carbon content of the activated biocarbon samples increased from 87.32 to 91.45 wt %. Moreover, the hydrogen content was from 0.79 to 0.91 wt %, for the nitrogen content was oscillated close to 1 wt %, and for the oxygen content from 6.62 to 10.77 wt %. Changes of the elemental composition result from the release of volatile substances during carbonization that resulted in the elimination of noncarbonaceous parts and enrichment of carbon element.^65^

Table 3 shows the data on the ash content and elemental composition of the prepared activated biocarbons based on biomass and agriculture raw material wastes according to X-ray fluorescence analysis.

The XRF results showed (Table 3) that activated biocarbons based on plant wastes contain small amounts of Mg, Si, P, K, Ca, and Fe. They were not fully removed after the carbonization and activation processes. They remained in trace amounts as a result of retention in the carbon matrix structure by various types of bonds (with the formation of chelate complexes and other interaction products). Kishibayev et al.^66,67^ received comparable results for activated biocarbons prepared from biomass waste.

Activated biocarbon GS (TC + SWVA) had the lowest ash content (0.62%), while birch char (TC + SWVA) (2.12%), PC char (HTC + SWVA) (2.72%), PC char (TC + SWVA) (4.72%), and CC (TC + SWVA) had the highest ash content (11.15%). The difference in the ash content of the obtained activated biocarbons is most likely due to the different types of plant materials used to produce the activated biocarbons.

**Figure 5:**
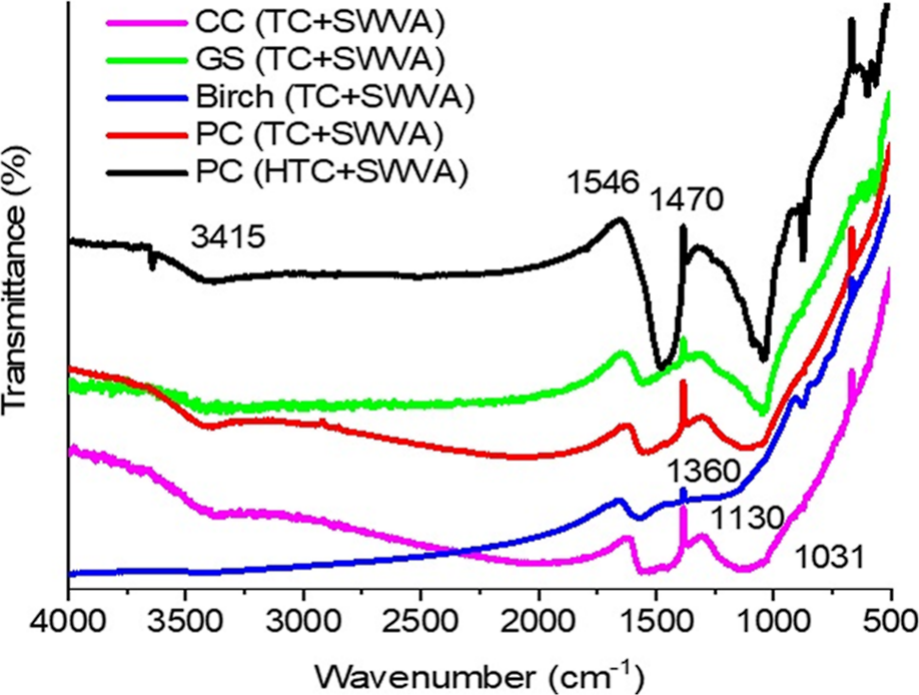
FTIR spectra of activated biocarbons.

The ash content of the activated biocarbons was examined to determine the purity of the samples. Ash is the residue left after the combustion process. The ash content tests the quality of activated biocarbons. High ash content leads to pore plugging and a decrease in surface area and pore volume.^68^ In addition, the content of silicon in the raw material affects the ash content. This means that the higher the content of silicon, the higher the ash content,^69^ which is confirmed by the presented results in Table 3. Figure 5 shows the results of Fourier transform infrared (FTIR) analysis of the activated biocarbons.

FTIR spectra have been investigated to qualitatively characterize functional groups on the surface of the activated biocarbon. The results of FTIR analysis of activated biocarbons obtained from various plant materials are shown in Figure 5. Peaks were determined for the samples in the range of wave numbers 500–4000 cm^–1^. The activated biocarbon spectra showed an absorption band at 3415 cm^–1^, a band at 1546 and 1470 cm^–1^, and an absorption band near 1360, 1130, and 1031 cm^–1^. The band at about 3415 cm^–1^ is attributed to the vibrations of *n*(OH) in the hydroxyl groups. Air atmosphere was used to record the spectra; therefore, an unambiguous interpretation of this band was difficult since it could come from both the −OH groups chemically bonded to the carbon surface and from the stretching vibrations of the H–O–H bonds of water molecules adsorbed on the carbon surface. Skeletal C=C vibrations in aromatic rings give two more bands at about 1546 and 1470 cm^–1^. These bonds correspond to the presence of oxygen–carbon compounds. These compounds affect the symmetry of the condensed system of aromatic rings so that the C=C bond becomes active in the infrared, showing absorption around 1600 cm^–1^. Thus, the bands at 1546 and 1470 cm^–1^ can be attributed to the carboxyl–carbonate structure.^47,70^ The band at 1360 cm^–1^ can be attributed to the *n*(C–O) vibrations in the carboxylate groups. Intense bands located in the region of 1130–1031 cm^–1^ can be assigned to C–O stretching vibrations in carboxyl, phenolic, and ether groups. Finally, the main oxygen groups present in activated biocarbons are the carboxylate groups and alcohol groups.^71^

Activated biocarbons are characterized by the chemical nature or surface chemistry more complex than the pore structure. Activated biocarbons are usually prepared from plant materials full of oxygen, so many of the functional groups of activated biocarbons contain oxygen atoms. The oxygen content can be increased in the production of activated biocarbon (during activation and/or carbonization) depending on the type of method used. Available carboxyl groups act as electron donors in the process of electrostatically oriented adsorption and confirm the possible ion exchange ability of physically activated adsorbents. The high content of carboxyl groups can be explained by the fact that activated biocarbon was synthesized at temperatures above 400 °C. Moreover, it was reported that an increase in the number of oxygen-containing surface functional groups increases the polarity of carbon surfaces, which, in turn, conduct to an increase in the selectivity of the carbon surface with respect to CO_2_^47,72^ and heavy metal ions.

**Figure 6:**
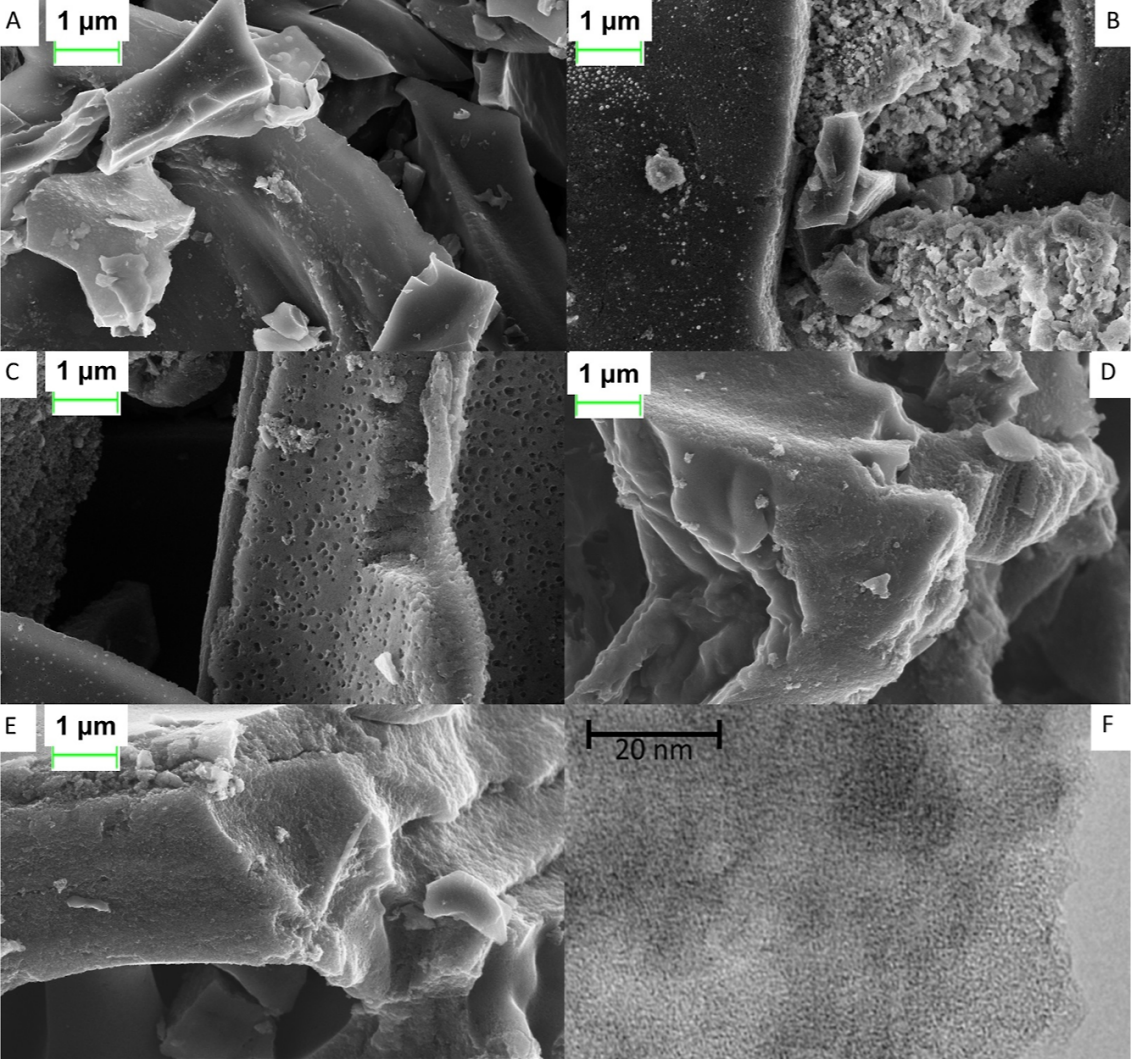
SEM images of activated biocarbons based on the waste of plant materials: (A) CC (TC + SWVA), (B) GS (TC + SWVA), (C) birch (TC + SWVA), (D) PC (TC + SWVA), (E) PC (HTC + SWVA), and (F) TEM image for the birch-activated biocarbon.

Scanning electron microscopy allows for a detailed examination of the surface morphology and particle size of activated carbons, aiding in understanding their physical properties and potential surface modifications. Scanning electron microscopy images show that the surface structure of the samples is different (Figure 6) and confirm the porosity of activated biocarbon based on the waste of plant materials. Analysis of microphotographs showed that the activated biocarbon on the basis of PC, GS, and CC prepared by thermal carbonization and activation with SWVA (Figure 6a,b,d) have a flaky surface with a developed porous structure. The birch-based activated biocarbon obtained by thermal carbonization and SWVA activation (Figure 6c) has a cellular surface with a developed porous structure. PC-based activated biocarbon after HTC (Figure 6e) had a loose and homogeneous surface with a developed porous structure. Differences in the pore sizes can be related to the decomposition of organic compounds during the process of activation and the nature of the carbon skeleton. A large amount of organic compounds in the samples after HTC, apparently, increases the number of pores created in the time of activation.^73^

The above results have shown that thermal, HTC, and activation by SWVA have a strong influence on the textural characteristics of activated biocarbons. The use of SWVA as an activator activates pores both on the surface of the sorbent and inside its structure with a high content of micropores, and the specific surface area of the resulting activated biocarbons increases several times.

The TEM image for the birch-activated biocarbon is shown in Figure 6F. The surface morphology of the birch is a porous multilayer texture, which is in accord with the *S*_BET_ results (Figure 1 and Table 1) that showed the formation of microporous carbonaceous materials along with the SEM results in Figure 6c.

### 3.2 Carbon Dioxide Adsorption Studies

Activated biocarbons have a high surface area and are commonly used as adsorbents for gases including carbon dioxide. The mechanism of carbon dioxide adsorption in activated biocarbons involves physical adsorption and chemisorption. Physical adsorption occurs when carbon dioxide molecules are attracted to the activated biocarbon surface due to weak van der Waals forces. This is typically the dominant mechanism of carbon dioxide adsorption in activated biocarbons at a low pressure and temperature. The high surface area of activated biocarbons provides a large number of sites with which carbon dioxide molecules can interact, resulting in high adsorption capacity. Chemisorption occurs when carbon dioxide molecules react with functional groups on the activated biocarbon surface. These functional groups may include hydroxyl (–OH), carboxylic acid (–COOH), or amine (–NH_2_) groups, which can form chemical bonds with carbon dioxide molecules. This mechanism of carbon dioxide adsorption is typically more important at higher pressures and temperatures and can lead to the irreversible adsorption of carbon dioxide on the activated biocarbon surface. The exact mechanisms of carbon dioxide adsorption in activated biocarbons can vary depending on the specific type of activated biocarbon and the conditions of the adsorption process. Factors such as PSD, surface chemistry, and temperature can all affect the adsorption behavior of carbon dioxide in activated biocarbons.^74,75^

Figure 8 shows the results of carbon dioxide sorption at 273 and 298 K and a pressure of 1 bar with activated biocarbons based on plant waste.

**Figure 7:**
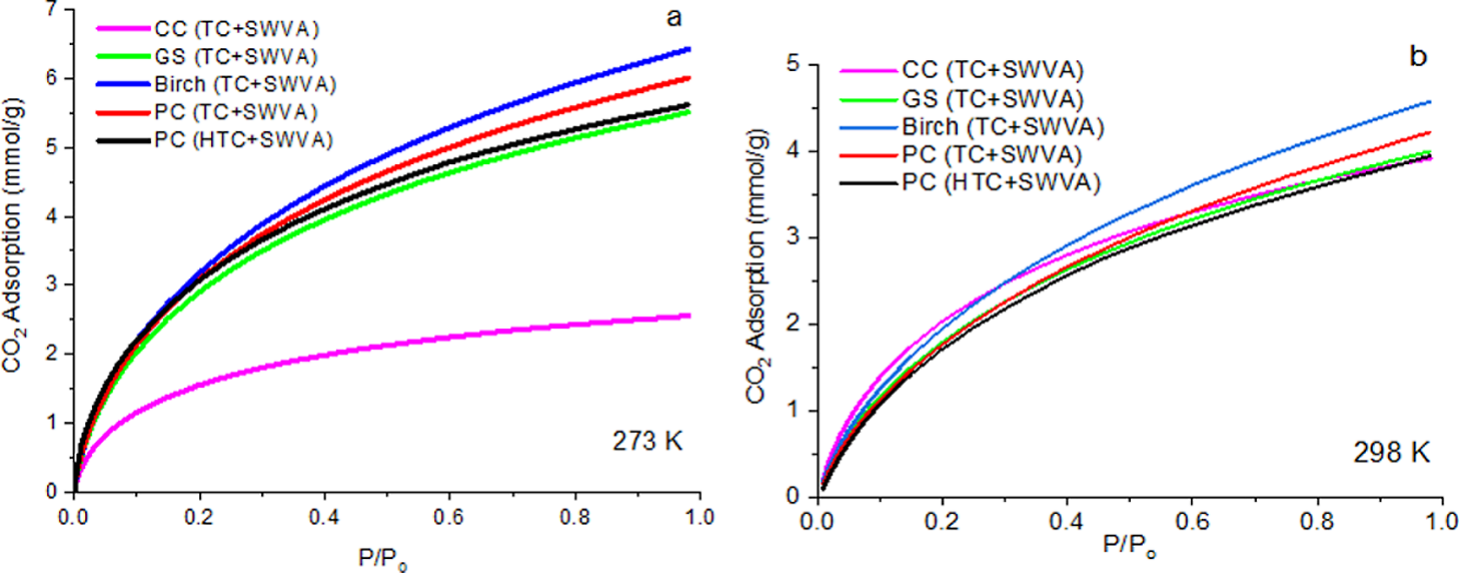
Isotherms of CO_2_ adsorption by activated biocarbons based on plant raw material waste at 1 bar and temperatures: (a) 273 and (b) 298 K.

All activated biocarbons were characterized with the same tendency. The adsorption capacity for CO_2_ increased according to pressure increase (Figure 7a,b). All CO_2_ isotherms correspond to type I isotherms according to IUPAC isotherm classification.^49^ Isotherm type I is typical for microporous activated biocarbons. For activated biocarbons made from biomass raw material waste, the carbon dioxide adsorption isotherms at 273 and 298 K have a similar course, rapidly increasing at low pressures and slowing down at higher ones. These isothermal similarities suggest that the adsorption mechanism was the same for all of the activated biocarbon samples tested. Based on Figure 7a, it was found that birch (TC + SWVA)- and PC (TC + SWVA)-activated biocarbons had the highest adsorption capacity for CO_2_ at 1 bar pressure and 273 K and were 6.43 and 6 mmol/g, respectively. The highest CO_2_ adsorption at 298 K and a pressure of 1 bar (Figure 7b) is observed on activated biocarbons based on birch (TC + SWVA) and PC (TC + SWVA) and is 4.57 and 4.22 mmol/g, respectively. The amount of CO_2_ adsorbed on activated biocarbons decreased with increasing temperature. This confirms the physical adsorption and exothermic nature of the CO_2_ sorption on activated biocarbons. A fundamental role in the interaction between CO_2_ and activated biocarbon is played by the van der Waals forces. At low temperatures, these molecular forces are stronger, however weaken at higher temperatures.^47^

The micropores were mainly used for the adsorption of CO_2_ on the obtained activated biocarbons through a micropore filling mechanism.^76^ This was because carbon dioxide molecules are adsorbed by micropores,^77^ and the amount of carbon dioxide sorbed on activated biocarbons is primarily due to the narrow volumes of micropores.^78^ Based on Figure 2b, it can be assumed that pores with a diameter of 0.3–1 nm are the most important for the sorption of carbon dioxide at 1 bar. Wickramaratne and Jaroniec,^79^ Grundy and Ye,^50^ and Li et al.^51^ proved that carbon dioxide sorption at 273 and 298 K and pressure 1 bar depends on micropores less than 1 nm in size.

Table 4 presents the comparative results of carbon dioxide sorption by activated biocarbons obtained from various carbon precursors.

**Table 4:** Sorption of CO_2_ by Various Activated Biocarbons at 1 bar, 273, and 298 K

	carbon dioxide sorption at 1 bar pressure [mmol/g]		
activated biocarbons	273 K	298 K	refs
rice husk	5.83	3.68	(80)
amazonian nutshells	5.14	3.67	(81)
nutshell		3.48	(82)
coffee grounds	3.60	2.40	(83)
coconut shell	5.60	3.90	(84)
bamboo	7.00	4.50	(85)
hazelnut shell	6.44	4.30	(86)
wood		2.90	(87)
andiroba shell	6.10	3.20	(88)
subabul sawdust		1.81	(89)
walnut shell	9.54	5.17	(65)
common oak leaves	6.20	5.40	(41)
surgical mask	3.90		(60)
petroleum coke	5.57	4.18	(90)
water chestnut shell	6.90	4.54	(91)
cupuassu shells	7.80	4.40	(92)
birch (TC + SWVA)	6.43	4.57	This work
PC (TC + SWVA)	6.00	4.22	This work

The results we obtained on CO_2_ adsorption with birch (TC + SWVA)- and PC (TC + SWVA)-activated biocarbons (Table 4) were quite good and can be competitive with other materials.

To analyze how textural parameters affect CO_2_ adsorption at 273 and 298 K and at 1 bar pressure determined the relationship between CO_2_ adsorption with respect to specific surface area (*S*_BET_), total pore volume (*V*_tot_), micropore volume for nitrogen (*V*_micN_2__), and micropore volume up to 1.4 nm for CO_2_ (*V*_micCO_2__) (Figure 9).

**Figure 8:**
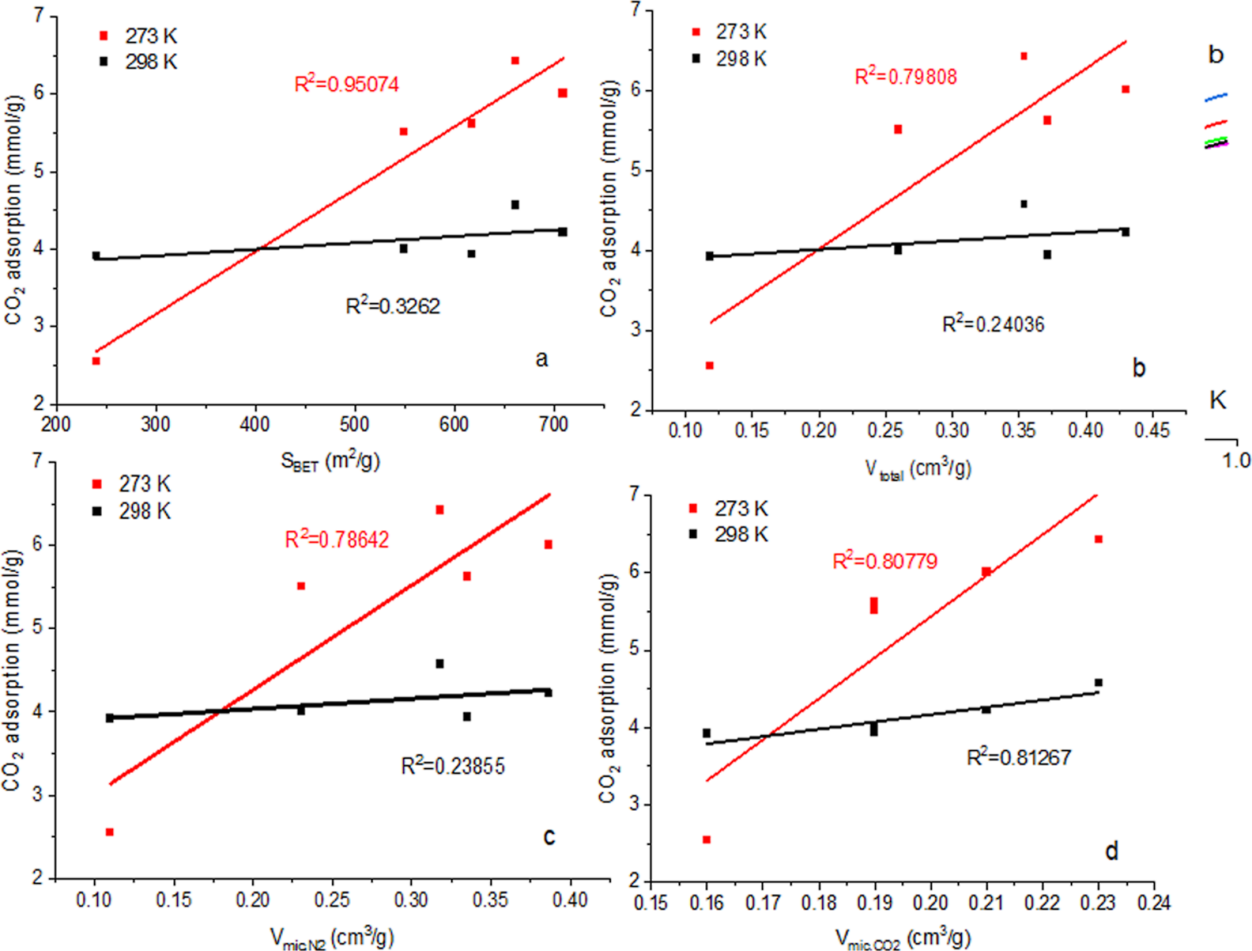
Carbon dioxide sorption at a pressure of 1 bar and at 273 and 298 K temperatures as a function of (a) *S*_BET_, (b) *V*_tot_, (c) *V*_micN_2__, and (d) *V*_micCO_2__

Figure 8 shows the effect of specific surface area (*S*_BET_) (Figure 8a), total pore volume (*V*_tot_) (Figure 8b), micropore volume determined from nitrogen sorption at 77 K (*V*_micN_2__) (Figure 8c), and micropore volume found by CO_2_ sorption at 273 K (*V*_micCO_2__) (Figure 8d) for carbon dioxide sorption. The coefficient of determination *R*^2^ is the proportion of the variance of the dependent variable that can be predicted from the independent variable. *R*^2^ equal to 1 means that the dependent variable can be predicted without an error from the independent variable. A small number of *R*^2^ values indicates no relationship between variables. Based on the values of *R*^2^ presented in Figure 8, conclusions were drawn about the influence of *S*_BET_, *V*_tot_, *V*_micN_2__, and *V*_micCO_2__ on carbon dioxide sorption.

**Figure 9:**
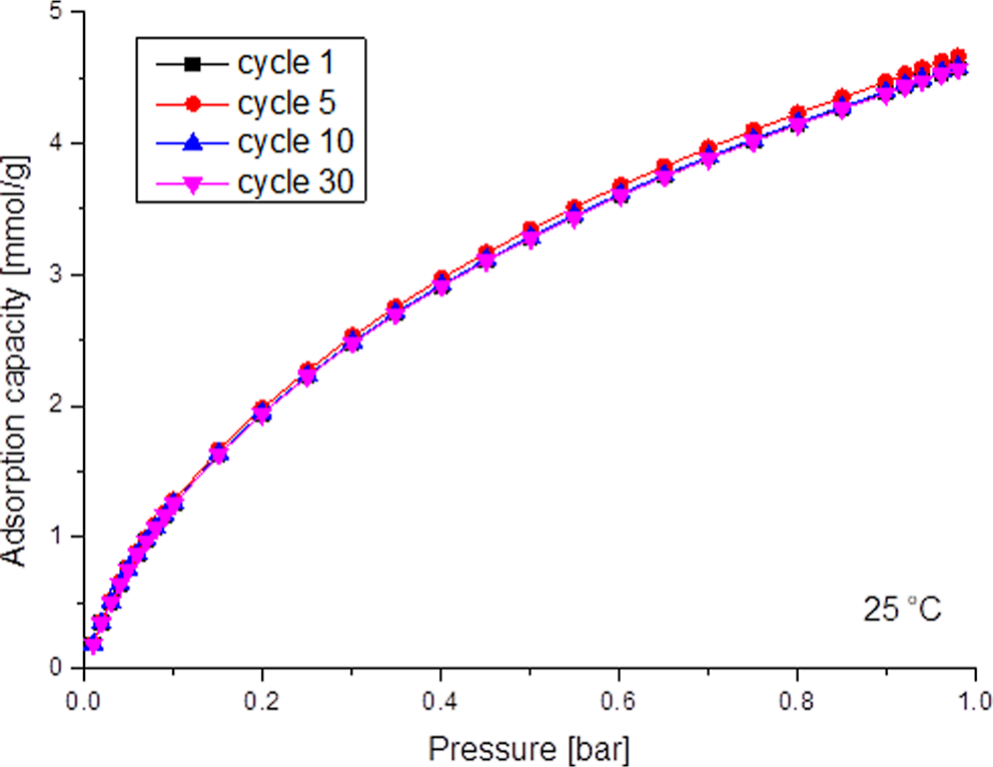
Multicycle CO_2_ adsorption isotherms for birch (TC-SWVA)-activated biocarbon at 25 °C up to 1 bar in 1st, 5th, 10^th^, and 30th cycles.

No corresponding relationship was found between carbon dioxide uptake at 273 K and total pore volume (*V*_total_) (Figure 8b) and micropore volume by nitrogen (*V*_micN_2__) (Figure 9c). However, the best correlation was obtained between carbon dioxide sorption at 273 K and specific surface area (*S*_BET_) (Figure 8a) and between micropore volume determined from CO_2_ sorption (*V*_micCO_2__) (Figure 8d). The dependence of carbon dioxide sorption at 298 K on specific surface area (*S*_BET_) (Figure 8a), total pore volume (*V*_tot_) (Figure 8b), and micropore volume by N_2_ adsorption (*V*_micN_2__) (Figure 8c) was not found. However, a high correlation was obtained between carbon dioxide sorption at 298 K and the micropore volume determined from carbon dioxide adsorption (*V*_micCO_2__) (Figure 8d). Based on Figure 2b, it was concluded that activated biocarbons contain micropores in the range of 0.3–1.1 nm, which were determined from measurements of carbon dioxide sorption at 273 K. Thus, the high adsorption of carbon dioxide at 273 and 298 K is due to the presence of micropores with a pore diameter from 0.3 to 1.1 nm determined by CO_2_ sorption (Figure 8d). And also, the high sorption of carbon dioxide at 273 K is connected with the specific surface area of the obtained activated biocarbons (Figure 8a). With an increase in the value of the specific surface area of the obtained activated biocarbons, the adsorption of CO_2_ at 273 K also increases (Figure 8a and Table 1).^60^

One of the important criteria for assessing the quality of a well-activated biocarbon as an adsorbent is its regenerative capacity. It determines the lifetime of the adsorbent, its reuse, and the total cost of the capture. To assess the possibility of easy regeneration and reuse of the material, the reversibility of CO_2_ adsorption was tested in 1, 5, 10, and 30 cycles at 25 °C up to 1 bar for the best sorbent birch (TC-SWVA) (Figure 9). Based on this measurement, it is possible to assess the regeneration capacity of activated biocarbons and, second, to verify the fact that CO_2_ does not bind to the sorbent material by chemisorption. In addition, it allows you to establish parameters that can serve as a reference point when trying to regenerate in real conditions on an industrial scale. As can be seen in Figure 9 after 30 cycles of CO_2_ adsorption, no changes were found. The highest standard deviation for the CO_2_ cycles was 0.06. The obtained results confirm the fact that the obtained activated biocarbon can be easily regenerated under mild conditions and that it retained its properties. Birch-activated biocarbon is a good sorbent in terms of its regeneration capacity.

**Figure 10:**
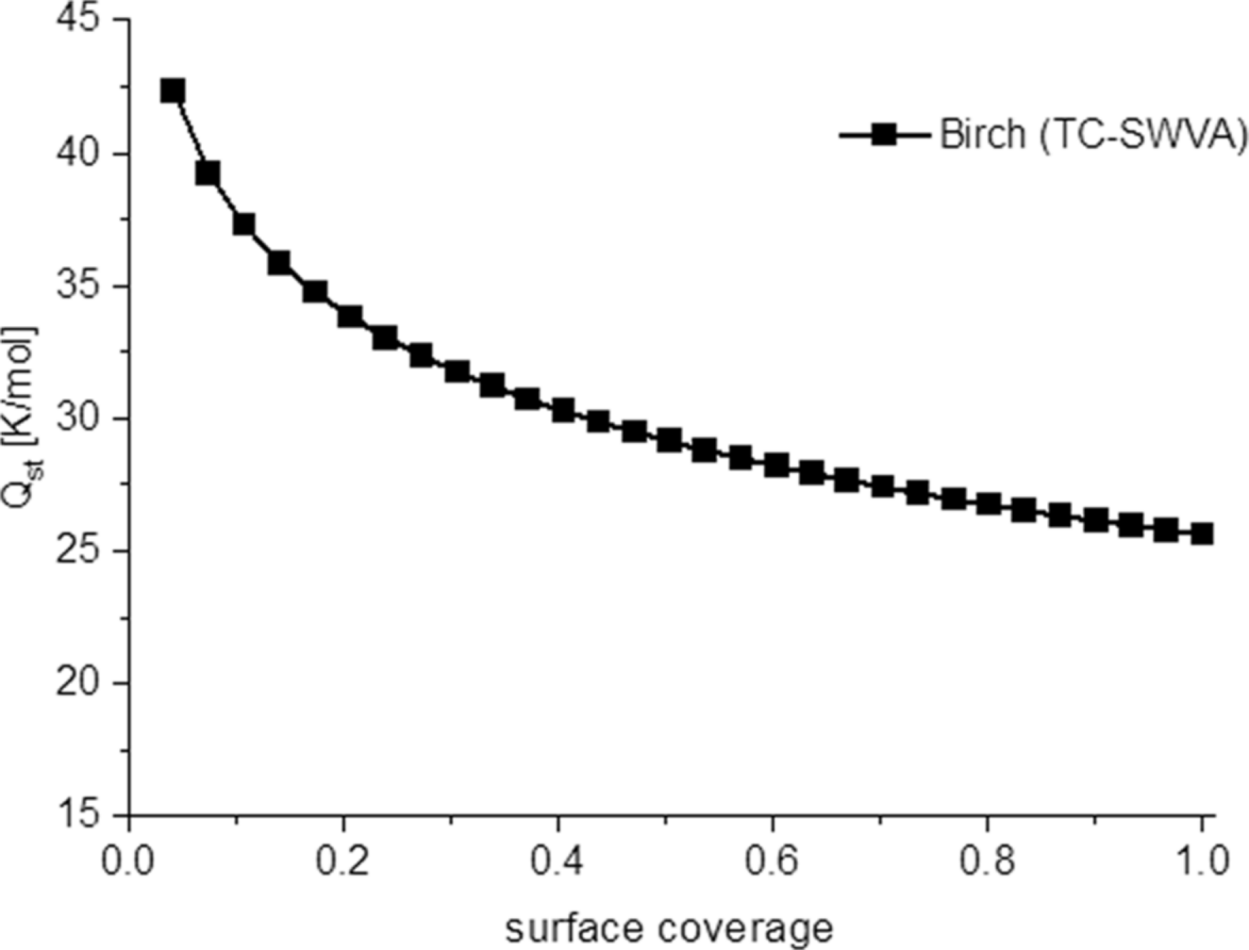
Isosteric heat of adsorption as a function of surface coverage for birch (TC-SWVA).

Figure 10 shows that the isosteric heat value of the adsorption (*Q*_iso_) is important to describe the interaction between the adsorbent and the absorbent. Information about the power of adsorption is provided. The higher values of adsorption isosteric heat show a stronger interaction between the adsorbate and adsorbent. High isosteric heat caused by adsorption causes high costs for regeneration. The surface coverage values of activated bicarbonate trichloride (TC-SWVA) adsorption heat range from 44 to 27 kJ/mol. The obtained values confirm the physical properties of CO_2_ sorption in the prepared activated biocarbons. The isosteric heat of adsorption decreased with coverage of the surface. Carbon dioxide is bound to the surface of activated carbon by the forces of van der Waals and can therefore be easily desorbed.^41^

### 3.3 Study of Heavy Metal Sorption

Activated biocarbon is also a commonly used adsorbent for the removal of metals from water due to its high surface area and strong affinity for various types of metal ions. The effectiveness of activated biocarbon for metal sorption is dependent on several factors, including the properties of the carbon itself, the properties of the metal ions, and the conditions under which adsorption occurs. One important factor affecting the performance of activated biocarbon for metal sorption is its pore structure. Activated biocarbon with a high surface area and a large number of micropores is generally more effective at adsorbing metal ions due to the increased surface area available for interaction. The presence of meso- and macropores can also improve the accessibility of metal ions to the adsorption sites on the carbon surface. The surface chemistry of the activated biocarbon also plays a significant role in metal sorption. The surface functional groups, such as carboxyl, hydroxyl, and phenolic groups, can act as binding sites for metal ions through ion exchange, chelation, and electrostatic interactions. The presence of these functional groups can be enhanced through chemical modification of the activated biocarbon surface, which can improve its effectiveness for metal sorption. The properties of the metal ions themselves also impact the performance of activated biocarbons for metal sorption. The size, charge, and chemical nature of the metal ions can affect their affinity for the carbon surface and the mechanisms by which they are adsorbed. For example, smaller metal ions with high charge densities tend to be more strongly adsorbed through electrostatic interactions, while larger metal ions may be more effectively removed through chelation or complexation. The conditions under which the adsorption occurs, such as pH, temperature, and the presence of competing ions, can also affect the effectiveness of activated biocarbons for metal sorption. In general, activated biocarbons are most effective for metal sorption at low pH values, where the surface functional groups are protonated and have a stronger affinity for metal ions. However, the pH range over which the activated biocarbon is effective depends on the specific functional groups present on the carbon surface. Overall, activated biocarbon is a versatile and effective adsorbent for the removal of metals from water. The effectiveness of activated biocarbon for metal sorption is dependent on several factors, including the properties of the carbon itself, the properties of the metal ions, and the conditions under which the adsorption occurs. Optimization of these factors can improve the effectiveness of activated biocarbon for metal sorption.^93^

#### 3.3.1 Effect of Initial pH and Isotherm from Aqueous Solution

It is important to previously carry out studies of the behavior of the pH of the solutions in investigations from aqueous solution, considering that each ionic species changes its speciation curve depending on this variable. That is why, it is a parameter that must be analyzed during this type of study on the adsorption of heavy metals on porous adsorbents.

In this research, pH behavior tests were carried out using the sample with the largest area and total volume, PC (TC + SWVA), as an adsorbent.

When analyzing the adsorption processes as a function of pH, the capacity varies, and this variation can be attributed to the chemical form of the metal ions, in particular when they form polyhydroxylated complexes.

When analyzing the behavior of the ions as a function of pH, at low pH values, the ions are found as M^2+^ and M(OH)^+^. This type of configuration can lead to effective competition between the H^+^ and H_3_O^+^ species. In this work, they were made for each metal ion. With these studies, some aspects were found in common for the ions studied here, and it is worth highlighting, for example, the behavior of Pb^2+^ and Zn^2+^. The respective speciation curves for these two ions can be made, for example, from aqueous solutions of Pb^2+^ and Zn^2+^ as a function of pH at 10 and 100 mg/L of the respective metal in the form of nitrate using the Visual MINTEQ computer program Version 2.30.^94^

**Figure 11:**
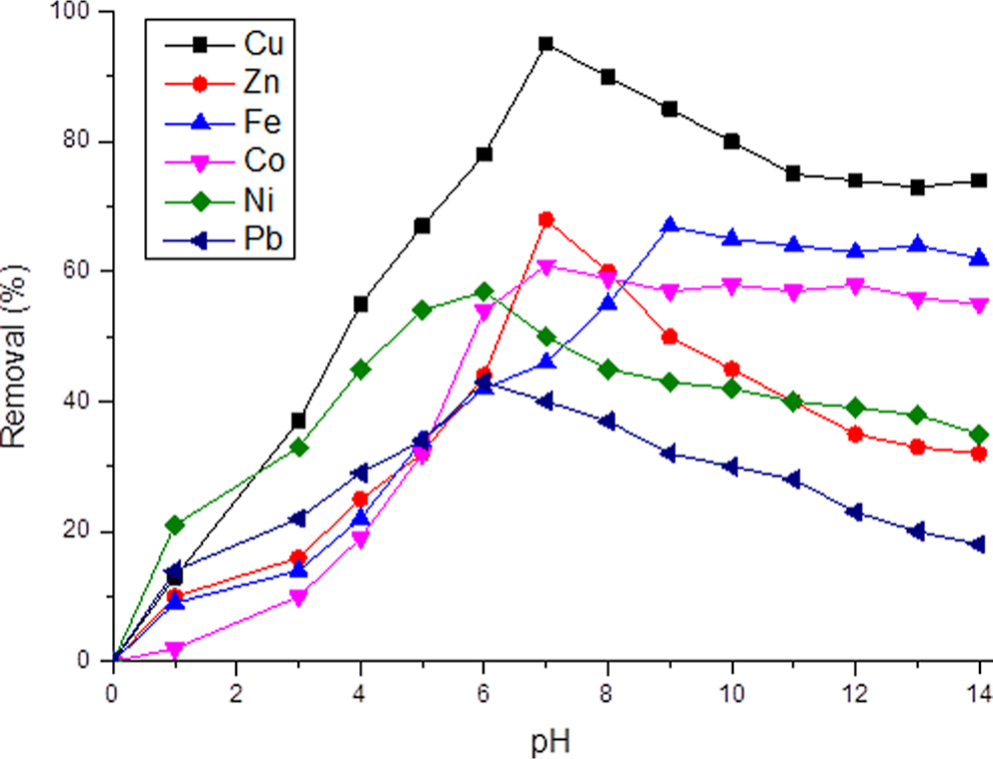
Effect of pH on the adsorption capacity of ions on activated biocarbon PC (TC + SWVA).

The integration of this type of calculation is very interesting because it allows clearly analyzing how the respective ions are found; for example, below pH 8.5, Pb occurs predominantly as Pb^2+^ and Pb(OH)^+^. In addition to the Pb^2+^ and Pb(OH)^+^ species, the nitrate species (PbNO)^+3^ is also present in a significant amount up to pH 6.5, and then its concentration starts, after which its concentration starts to decrease. It was detected in this type of analysis that species such as Pb^2+^, (Pb(NO_3_)_2_(aq), Pb_4_(OH)_4_^+4^, and (Pb_2_(OH)_3_^+^) are also found in aqueous solution, but in the entire range of pH, its concentration does not change significantly. In the case of the Zn ion, it is formally found as Zn^2+^. The concentration of Zn^2+^ begins to decrease after pH ≈ 7.5 in the systems tested in this investigation. Other types of species may occur as Zn (ZnNO^+3^, Zn(NO_3_)_2(aq)_, Zn(OH)^−^_3_, Zn(OH)_2_^–4^, and Zn_2_(OH)_3_^+^) are present but in negligible concentrations under solution conditions of our experiments. On the other hand, taking into account the functional groups that are generated on the surface of the adsorbent during its preparation and taking into account that this depends on the different conditions under which they were obtained, which causes different active groups to be generated, which become specific adsorption sites at a given time, these become more sensitive to the adsorption of ion-adsorbent groups of solids with the variation of pH. The studies to determine the effect of the pH variable on the adsorption of Pb^2+^ and Zn^2+^ ions, in equilibrium at different pH values, were carried out in the pH range 2–12 for the two metals (Figure 11), obtaining as a result that maximum adsorption of lead ions by each activated biocarbon from aqueous solution was observed at pH 6.0 and maximum adsorption of zinc ions occurred at pH 7.0. Examining the scientific literature, it is found that several researchers^95−97^ established a similar behavior using other types of adsorbents: maximum absorption at pH close to 6.0. This can be interpreted as a pH close to 6.0, implying that at higher concentrations of H^+^, the surface of the adsorbent becomes more positively charged, which reduces the attraction between the adsorbent and the metal ions. On the other hand, as the pH increases, more negative charges are found on the surface, which facilitates the adsorption retention of positively charged ions.^98^ At higher pH values, the lead and zinc ions will precipitate as hydroxides, which generates a decrease in the adsorption rate and, subsequently, the percentage of removal of metal ions. On the other hand, if the case of the adsorption of the copper ion is analyzed, the absorption and the percentage of elimination of this metal from aqueous solution, it is observed that it is strongly affected by the pH variable; obtained results are presented in Figure 11. The results show that the adsorption of copper increases between pH 1 and 7. If analyzed in detail, the adsorption for this metal increases significantly between pH 2 and 7, reaching a maximum capacity at pH 7 with 95% removal of the metal. At higher pH values, the adsorption capacity decreases slightly in the pH range of 8–10. The lowest adsorption capacity occurs at low pH’s (pH = 1), which is probably due to the fact that the higher mobility of the H^+^ ions present favored the preferential adsorption of hydrogen ions compared to Cu^2+^ ions. As suggested by some authors, it can be suggested that at low pHs, the surface of the adsorbent is surrounded by hydrogen ions (H^+^), which means that metal ions cannot approach the adsorbent sites. This means that at higher concentrations of H^+^, the surface of the adsorbent becomes more positively charged so that the attraction between adsorbent and metal cations is reduced.

When carrying out experiments on the sorption of heavy metals at different pH values (Figure 11), we used activated biocarbon PC (TC + SWVA) because this activated biocarbon had a high specific surface area and porosity.

It is therefore worth noting that if the pH is increased, negative charges will be generated on the surface of the adsorbent, which will favor the elimination of the copper ion by means of the adsorption process. It can then be generalized that the adsorption of metal cations increases as a function of pH variation due to the instability of these in aqueous solution. However, it should be pointed out that at extreme pH values (pH 9, 10, 11, and 12), there is normally a decrease in adsorption capacity. This phenomenon can be attributed to the precipitation of the ions, as in the case of the copper ion; this is associated with the solubility constants (*K*_sp_). If you look carefully at pH 6, there are three species present in the solution, as suggested by some authors in the scientific literature, Cu^2+^ in very small quantities and Cu(OH)^+^ and Cu(OH)_2_ in large quantities. These three species can be adsorbed on the adsorbent through an ion exchange mechanism, which occurs with the groups that are on the surface of each of the adsorbents prepared in this work and through hydrogen bonds. The adsorption of the cobalt ion depending on the pH shows a strong removal of this ion depending on the pH: it removes strongly between 1 and 7, and then from this pH, the removal% remains constant. This strong adsorption at the beginning can be associated with the different p*K*_a_ and p*K*_b_ values of the functional groups developed on the activated biocarbon surface. The results of the adsorption capacity of *C*_o_ vs pH are shown in Figure 11. It was found that the removal of Co^2+^ at strong acidic pH values was very low [24% with PC (TC + SWVA)]. However, with the pH increase from 2 to 5, the elimination of Co^2+^ was increased in a buffered way for this sample (PC (TC + SWVA)); for the other samples also analyzed, for example, removal of 80% was achieved, the highest (TC + SWVA), 87.5%, and PC (HTC + SWVA)], 92% PC (TC + SWVA). Here, it is worth noting that the effect of pH can be explained considering the concept of pH at the point of zero charges (pH_pzc_) and the cobalt speciation curve in the solution with a pH in the range 2–6, where Co is mainly present as Co^2+^.^99,100^ Above pH_pzc_, the surface charge of the adsorbent is negative, and below pH, it will be positive. For this particular case of cobalt, the amount of adsorption above pH_pzc_ was higher, which is due to the interaction between the Co^2+^ ion with the negatively charged surface groups of the activated biocarbon samples. On the other hand, at low pH, especially below pH_pcz_, the positively charged Co^2+^ species can probably generate a repulsion situation on the carbon surface that has the same charge and, therefore, it results in a decrease in the Co^2+^ adsorption.

Finally, and to summarize the adsorption behavior of the cognate as a function of pH, what is generally observed with a variation of the adsorption capacity of cobalt as a function of pH may be due to the low generation of cobalt complexes. It is probable that at low pH values in aqueous solutions, cobalt can be protonated with some functional groups (or they are difficult to dissociate).

With respect to iron, the results as a function of pH show that this variable influences its adsorption capacity, which makes it an important parameter to study when conducting adsorption capacity studies is required. Iron presents a graph similar to that of cobalt (see Figure 11); however, it is worth mentioning that it is more cushioning throughout its journey in a first journey between pHs from 1 to 5, its removal capacity increases, and then between 5 and 9, it retakes a strong increase, until reaching *a*% removal of 70% for the sample of activated biocarbon PC (TC + SWVA); these types of results are in very good agreement with other cases reported in the specialized literature.^101−103^ In other words, it can be pointed out for the iron ion according to the results shown in Figure 11 that by increasing the pH, the adsorption capacity increases to a maximum value of 9, a value beyond which the adsorption capacity becomes practically constant. Finally, in this case, it can also be mentioned that the adsorption efficiency of Fe^2+^ on activated biocarbon can also be described on the conceptual basis of pH_pzc_. As mentioned above, below pH_pzc_, the activated biocarbon surface is positively charged, resulting in competition between H^+^ ions and dye cations to reach the surface. Eventually, the active sites on the biocarbon surface will be surrounded mainly by H^+^ ions and to some extent by dye molecules.^99,100^ In summary, several aspects can be generalized regarding the analysis of the removal efficiency of heavy metal ions on samples of activated biocarbon; initially, it is worth highlighting that the H^+^ ions within the adsorption mechanism can limit the interactions between the molecules corresponding to the cationic groups (MB^+^) and the surface of the activated biocarbon. Repulsive thermodynamic forces make contact between positively charged groups on the surface of activated biocarbon difficult and thus contribute to the mechanism of the adsorption capacity of iron adsorbed on activated biocarbon at pH < 4 (140 mg/g). The fact is highlighted that, subsequently, an amount of iron adsorbed on activated biocarbon at pH > 4 is presented. The maximum amount adsorbed remains almost unchanged in the pH range of 7–10 since there were no significant changes in the amount of iron adsorbed on activated biocarbon.^99−101^ Therefore, it is ratified that the pH of the solution has a significant impact on the adsorption of heavy metals from aqueous media by a specific adsorbent. Finally, the variations in the percentage of Ni^2+^ removal at different initial pH values (2–12) are shown in Figure 11. The trend showed a gradual increase in Ni^2+^ absorption from 27.1 to 58.9% with an increase in the pH from 3 to 6. Subsequently, a small decrease in the adsorption capacity is observed between a pH of 6–8 and then remains constant. This behavior is based on the above arguments considering that this metal ion also has a 2^+^ charge. At lower pH values, hydrogen ions are in higher concentration and compete for the sorption site present on the adsorbent surface with metal ions, but with increasing pH, the presence of hydroxyl ions in the solution provides more sorption sites. Sorption for metal ions occurs by deprotonating the adsorbent surface; therefore, more metal ions are adsorbed.^104^

#### 3.3.2 Adsorption Isotherms of Pb^2+^, Zn^2+^, Fe^2+^, Ni^2+^, Co^2+^, and Cu^2+^ from Aqueous Solution

Within the investigations of adsorption from aqueous solution, it is important to adjust the results taken from the experiments to the different mathematical models found in the scientific literature. This is very important because they allow an adequate interpretation of the adsorption phenomenon between the adsorbate and the adsorbent, among other aspects, allowing the determination of the respective adsorption capacities. These models also provide, according to the approach of each of them, how the adsorption between the respective ions and activated biocarbons takes place. Additionally, they allow one to show the efficiency of an adsorbent and also to estimate the economic feasibility of commercial applications of the biocarbons for the specified solute. For the analysis of the obtained results, the Langmuir, Freundlich, Sips equations have been adjusted, and their equations are represented in eqs 3–7.

Langmuir Isotherm
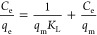
3

**Figure 12:**
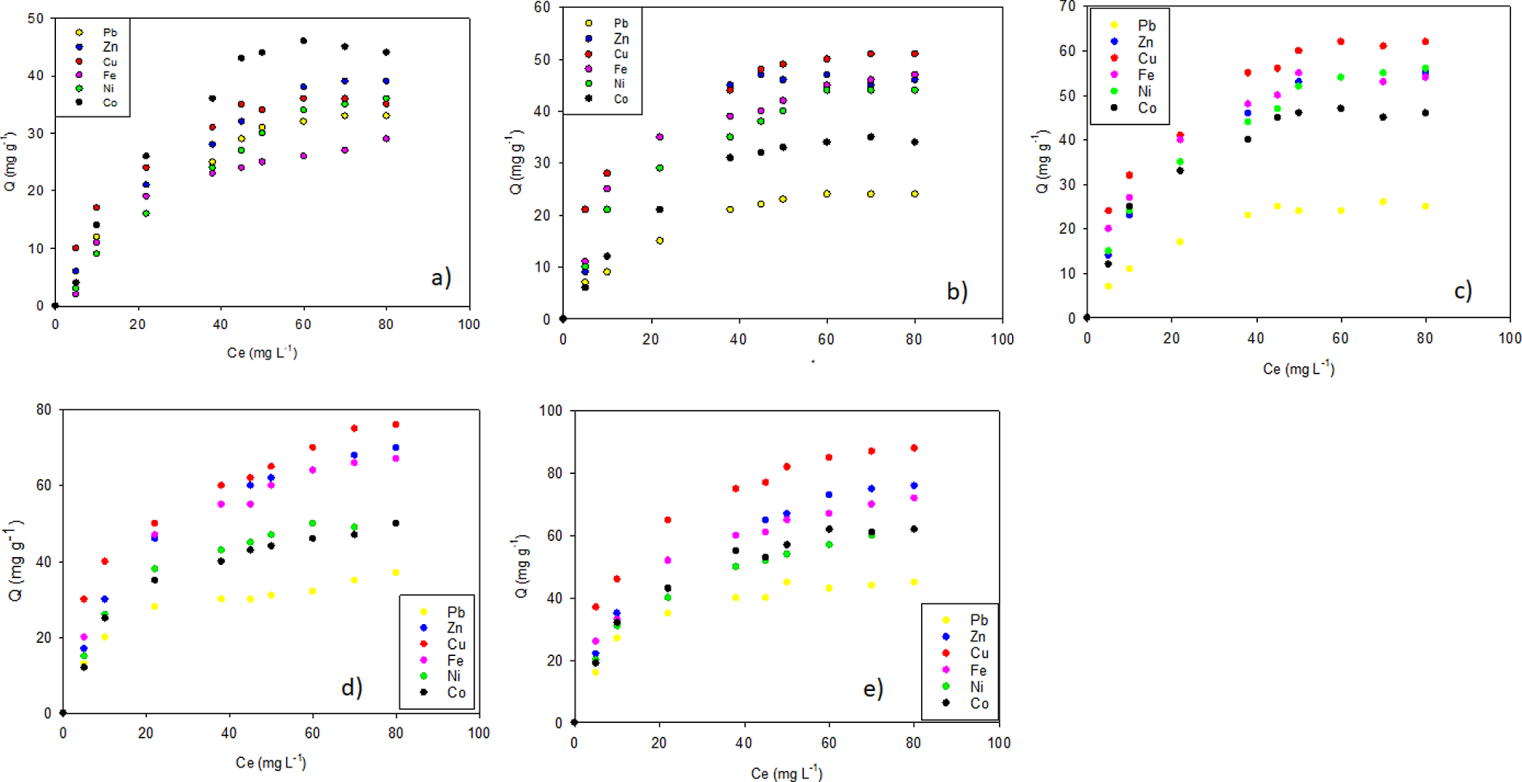
Freundlich, Langmuir, and Sips adsorption isotherms of ions onto (a) CC (TC + SWVA); (b) GS (TC + SWVA); (c) PC (HTC + SWVA); (d) birch (TC + SWVA); and (e) PC (TC + SWVA) from aqueous solution: adsorption order: Co^2+^ > Zn^2+^ > Cu^2+^ and Ni^2+^ > Pb^2+^> Fe^2+^

Freundlich Isotherm
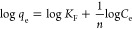
4*q*_e_ is the equilibrium quantity of ions adsorbed on activated biocarbons prepared in this research (mg/g), *C*_e_ is the equilibrium concentration of ions in solution (mg/L), and *K*_F_ (L/g) and *n* are Freundlich adsorption constants. Meanwhile, *q*_m_ is the maximum adsorption quantity (mg/g) of the adsorbent, and *K*_L_ is the Langmuir adsorption constant (L/mg). The Freundlich model explains the adsorption of the heterogeneous system and reversible adsorption. On the other hand, the Langmuir adsorption isotherm described the adsorption on homogeneous sites inside the adsorbent. Monolayer adsorption is described using Langmuir’s equation.^105^ The Langmuir isotherm characteristic was evaluated using the equilibrium parameter (*R*_L_),^105^ as defined in eq 5.
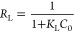
5where *K*_L_ (L/mg) is the Langmuir constant and *C*_o_ is the initial metal concentration (mg/L). The *R*_L_ value ranged from 0 to 1. The value of *R*_L_ determines the nature of the isotherm. *R*_L_ > 1 indicates unfavorable adsorption, while the 0 < 1 indicates favorable adsorption. Adsorption is irreversible at *R*_L_ = 0 and linear at *R*_L_ = 1. Figure 12a–e shows the respective adsorptions’ isotherms of the respective ions adjusted to these models.

The Sips adsorption isotherm model (also known as the Langmuir–Freundlich model) combines the Freundlich isotherm model and the Langmuir isotherm model.^106^ The Sips isotherm model (see eqs 5 and 6) was used to predict the heterogeneity of the adsorption system. If *n*_s_ = 1 or close to 1, it indicates that the system is homogeneous. *K*_s_ (L/g) is associated with adsorption energy.^106,107^
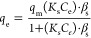
6

The linearized form is given as follows
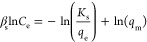
7where *q*_e_ (mg/g) is the quantity of ions mount adsorbed at equilibrium, *C*_e_ (mg/L) is the concentration of adsorbate at equilibrium, *q*_m_ (mg/g) is the maximum adsorption capacity of Sips, and β_S_ is the Sips exponent (dimensionless). *K*_S_ is the Sips equilibrium constant which is related to the characteristic energy of the micropores, and parameter β_s_ varies with the degree of heterogeneity of the adsorbent surface. When β_s_ is greater than 1, the phenomenon of positive cooperation between the adsorbent and the adsorbate is established, while for a value less than 1, this positive cooperation does not exist. A value equal to 1 assumes Langmuir-type behavior.^108^

Sips suggested an equation that combines the Freundlich and Langmuir isotherms after recognizing the issue of a continual rise in the adsorbed quantity with an increase in concentration in the Freundlich equation. This results in an equation that shows a finite limit at high concentration.^109^ The Sips model is the most appropriate, with three parametric isotherms used for monolayer adsorption studies. It can be used for heterogeneous systems and is valid for localized adsorption without adsorbate–adsorbate interactions.^109^ The Sips model does not follow Henry’s law at low concentrations of adsorbate because it approaches the Freundlich isotherm. On the other hand, it shows the monolayer adsorption behavior of the Langmuir model at a high concentration. The parameters of the model are controlled by the temperature, pH, and change in concentration. With a single parameter, the most basic isotherm is Henry’s model, applicable at low deadsorbate concentrations only. Monolayer adsorption on homogeneous sites and multilayer adsorption on heterogeneous sites can be modeled using Langmuir and Freundlich adsorption, respectively. Both adsorption isotherms involve two parameters. The Freundlich, Langmuir, and Sips model graphs (Figure 12a–e) are plotted based on the experimental results. The adsorption isotherm parameters were determined based on the intercept and slope of the three models’ equations plotted with their equations in the linear form.^110^ Constant parameters and correlation coefficients calculated for Freundlich, Langmuir, and Sips isotherm models are summarized in Table 5.

**Table 5:** Adsorption Isotherm Parameter Analysis of Ions onto Activated Biocarbons from Aqueous Solutions at 25 °C

CC (TC + SWVA)					
	Langmuir	*q*_m_ (mg/g)	*K*_L_ (mL/mg)	*R*_L_	*R*^2^
Co		55.65	0.2354	0.021	0.9893
Zn		38.46	0.3212	0.019	0.9821
Cu		37.64	0.2787	0.032	0.9811
Ni		34.42	0.3174	0.002	0.9787
Pb		31.32	0.2932	0.023	0.9823
Fe		28.57	0.3422	0.034	0.9856
	Freundlich	*q*_m_ (mg/g)	*K*_F_ (mol–1 mg–1)	1/*n*	*R*^2^
Co		N/A	50.00	0.574	0.9452
Zn		N/A	26.76	0.491	0.9832
Cu		N/A	24.21	0.489	0.9712
Ni		N/A	22.23	0.519	0.9573
Pb		N/A	20.19	0.561	0.9575
Fe		N/A	18.23	0.479	0.9568
	Sips	*q*_m_ (mg/g)	*K*_s_ (mL/mg)	β_s_	*R*^2^
Co		66.75	0.0212	1.133	0.9949
Zn		52.12	0.0330	1.023	0.9958
Cu		49.21	0.0443	1.130	0.9927
Ni		47.23	0.0492	1.379	0,9919
Pb		42.34	0.0432	1.460	0.9909
Fe		40.21	0.0492	1.260	0.9855

GS (TC + SWVA)					
	Langmuir	*q*_m_ (mg/g)	*K*_L_ (g/mol)	*R*_L_	*R*^2^
Cu		52.02	0.0454	0.002	0.9912
Zn		47.45	0.0583	0.032	0.9745
Fe		45.67	0.0915	0.012	0.9895
Ni		40.65	0.0698	0.129	0.9873
Co		33.76	0.0509	0.231	0.9931
Pb		21.70	0.0365	0.234	0.9862
	Freundlich	*q*_m_ (mg/g)	*K*_F_ (mol–^1^ mg–^1^)	1/*n*	*R*^2^
Cu		N/A	50.23	0.434	0.9732
Zn		N/A	44.34	0.395	0.9213
Fe		N/A	40.23	0.315	0.9884
Ni		N/A	36.46	0.371	0.9664
Co		N/A	29.45	0.423	0.9795
Pb		N/A	26.45	0.485	0.9543
	Sips	*q*_m_ (mg/g)	*K*_s_ (mL/mg)	β_s_	*R*^2^
Cu		73.32	0.048	1.042	0.9992
Zn		65.34	0.085	1.814	0.9948
Fe		60.12	0.044	1.345	0.9948
Ni		55.43	0.085	1.223	0.9913
Co		50.34	0.047	1.453	0.9935
Pb		39.70	0.056	1.324	0.9946

PC (HTC + SWVA)					
	Langmuir	*q*_m_ (mg/g)	*K*_L_ (g/mol)	*R*_L_	*R*^2^
Cu		68.98	0.0568	0.014	0.9887
Fe		56.86	0.0438	0.034	0.9933
Ni		55.67	0.0794	0.344	0.9872
Zn		54.88	0.0806	0.168	0.9924
Co		47.67	0.0489	0.543	0.9952
Pb		29.87	0.0694	0.321	0.9872
	Freundlich	*q*_m_ (mg/g)	*K*_F_ (mol–^1^ mg–^1^)	1/*n*	*R*^2^
Cu		N/A	65.32	0.398	0.9612
Fe		N/A	52.65	0.444	0.9802
Ni		N/A	50.34	0.338	0.9841
Zn		N/A	46.32	0.334	0.9734
Co		N/A	43.87	0.429	0.9885
Pb		N/A	24.43	0.365	0.9565
	Sips	*q*_m_ (mg/g)	*K*_s_ (mL/mg)	β_s_	*R*^2^
Cu		76.45	0.068	1.476	0.9980
Fe		63.43	0.395	1.276	0.9973
Ni		62.12	0.044	1.165	0.9991
Zn		58.45	0.078	1.033	0.9982
Co		52.65	0.032	1.015	0.9987
Pb		34.87	0.084	1.026	0.9899

Birch (TC + SWVA)					
	Langmuir	*q*_m_ (mg/g)	*K*_L_ (g/mol)	*R*_L_	*R*^2^
Cu		81.98	0.0102	0.001	0.9862
Zn		68.45	0.0232	0.123	0.9812
Fe		63.41	0.0188	0.001	0.9799
Ni		46.66	0.0420	0.023	0.9919
Co		42.76	0.0662	0.043	0.9979
Pb		36.17	0.0658	0.013	0.9949
	Freundlich	*q*_m_ (mg/g)	*K*_F_ (mol–^1^ mg–^1^)	1/*n*	*R*^2^
Cu		N/A	77.34	0.3059	0.9816
Zn		N/A	62.23	0.4232	0.9869
Fe		N/A	58.49	0.3295	0.9988
Ni		N/A	42.43	0.4144	0.9818
Co		N/A	38.87	0.3493	0.9735
Pb		N/A	33.21	0.3812	0.9767
	Sips	*q*_m_ (mg/g)	*K*_s_ (mL/mg)	β_s_	*R*^2^
Cu		95.12	0.074	1.238	0.9987
Zn		79.45	0.046	1.435	0.9938
Fe		70.23	0.054	1.345	0.9988
Ni		56.34	0.044	1.291	0.9932
Co		51.43	0.086	1.123	0.9978
Pb		43.98	0.072	1.108	0.9984

PC (TC + SWVA)					
	Langmuir	*q*_m_ (mg/g)	*K*_L_ (g/mol)	*R*_L_	*R*^2^
Cu		96.45	0.1046	0.007	0.9937
Zn		67.88	0,0609	0.145	0.9966
Fe		65.77	0.0977	0.043	0.9936
Co		60.70	0.0768	0.066	0.9945
Ni		58.57	0.0708	0.012	0.9935
Pb		42.66	0.0716	0.083	0.9944
	Freundlich	*q*_m_ (mg/g)	*K*_F_ (mol–^1^ mg–^1^)	1/*n*	*R*^2^
Cu		N/A	91.56	0.297	0.9708
Zn		N/A	62.32	0.393	0.9902
Fe		N/A	59.23	0.307	0.9931
Co		N/A	55.98	0.348	0.9892
Ni		N/A	51.98	0.366	0.9951
Pb		N/A	47.65	0.361	0.9802
	Sips	*q*_m_ (mg/g)	*K*_s_ (mL/mg)	β_s_	*R*^2^
Cu		110.2	0.111	1.353	0.9948
Zn		78.45	0.039	1.045	0.9989
Fe		71.34	0.076	1.321	0.9987
Co		68.33	0.043	1.453	0.9995
Ni		60.12	0.066	1.045	0.9978
Pb		52.12	0.065	1.066	0.9948

Based on the values of the regression coefficient presented in Table 5, the Sips model was found to be the best-fitted model with *R*^2^ more than 0.99 for all adsorbents and metal ions studied (*R*^2^ = 0.9788). The CC (TC + SWVA) sample, despite the fact that its adsorption capacity compared to the other adsorbents was not the highest, presented an acceptable *R*^2^ for the Langmuir model after the Sips model, with correlation coefficients for the Co^2+^ ion between 0.9853 and 0.9811 for the Langmuir model. A more detailed analysis of the adsorption capacity of each of the ions is studied on the activated biocarbons prepared in this research, it was possible to establish that the order of adsorption capacity is as follows: PC (TC + SWVA)> Birch (TC + SWVA) > PC (HTC + SWVA) > GS (TC + SWVA) > CC (TC + SWVA).

In the case of PC (TC + SWVA) biocarbon, it managed to adsorb a greater amount for all the ions studied, greatly surpassing materials prepared and reported in the literature. For the Cu^2+^ ion, for example, it managed to retain more than 96% (wt/wt), followed by Zn^2+^ (68%), Fe^2+^ (65%), Co^2+^ (60%), Ni^2+^ (59%), and Pb^2+^ (43%). The fit model for this biocarbon shows, as mentioned before, that it best fits the three-parameter model, that is, the Sips model, where its *R*^2^ ranged between 0.9899 and 0.9991. The βs values for adsorption for all ions were found to be greater than 1, which means that the phenomenon of positive cooperation between the adsorbent and the adsorbate is established. As mentioned before, the Sips model is a combination of the Langmuir and Freundlich models; if the *R*^2^ values of the adjustment to the Freundlich models for these biocarbons are observed, they are very acceptable, which indicates that at a given moment, it could be said that in a good concentration range, the ions are adsorbed adjusting to this model. Additionally, the value obtained for *R*_L_ is less than 1, which indicates that the adsorption process for the adsorbed ions by the adsorbent is favored.

In summary, furthermore, the value of *R*_L_ under 1 (Table 5) indicates that the ion adsorption process occurs in a homogeneous system and is likely to also follow the Langmuir model.^110^ Therefore, the ion adsorption in adsorbents studied here is more likely to follow the Langmuir model. According to the Langmuir isotherm model, the ion adsorption process takes place on the homogeneous surface of adsorbents. The model also postulated that each ion is uniformly distributed on the active site of activated biocarbons under research. Furthermore, these data support the hypothesis that ion adsorption on activated biocarbons occurs via monolayer adsorption. From the calculation of the nonlinear Sips isotherm model, it was obtained that the maximum adsorption capacity of the activated biocarbons in this study was higher than that of the other adsorbents. This demonstrates the potential of modified activated biocarbons as an effective adsorbent to remove ions from aqueous solutions. The *R*_L_ value less than 1 was obtained at 25 °C (Table 5), which implies that the adsorption of ions on the adsorbents is favorable at the investigated temperature. In the parameters of the Freundlich isotherm model, *K*_F_ and 1/*n* (dimensionless) represented the adsorption capacity and intensity, respectively. The value of the constant *n* in the Freundlich equation corresponds to the feasibility of adsorption, where the value of 1/*n* < 1 indicates a more favorable adsorption process,^105−107^ which was present in all the adsorption processes studied in this investigation.

The results of this research show that both the starting materials and the preparation process of activated biocarbons influence not only their textural characteristics but also their chemical properties, developing different functional groups that allow them to be very promising adsorbents and additionally that have broad adsorption spectrum for the tested ions. It is clear that all of the adsorbents presented here have a favorable affinity during the adsorption process and in a wide range.

## 4 Conclusions

In conclusion, we have demonstrated that the narrow microporosity-activated biocarbons from waste biomass (CC, GS, birch, and PC) obtained by thermal and HTC and activation with SWVA exhibit excellent properties as sorbents for CO_2_ capture and heavy metal sorption. The obtained results of the study confirm the production of highly porous activated biocarbons. The specific surface area of the obtained activated biocarbons was 240–709 m^2^/g, and the total pore volume was from 0.12 to 0.43 cm^3^/g. The percentage of microporosity of activated biocarbons was 89–92%, of which the best activated biocarbons based on birch and PC obtained by thermal carbonization and activation with SWVA had a microporosity of 90%. FTIR analysis showed that carboxyl and alcohol groups are present on the surfaces of activated biocarbons. SEM microscopy of the obtained biocarbons showed a flaky, cellular, and homogeneous surface with a developed microporous structure. XRD analysis proved the turbostratic structure of the obtained activated biocarbons, and Raman spectrometry confirmed the XRD data. According to the results of XRF analysis, it was found that activated biocarbons based on plant raw material waste contain significant amounts of Mg, Si, P, K, Ca, and Fe. The ash content of the obtained activated biocarbons was in the range from 0.62 to 11.15%, and the ash content is influenced by the silicon content in the raw material; the higher the silicon content, the higher the ash content. The adsorption of CO_2_ and heavy metals on the obtained activated biocarbons was studied. Activated biocarbons based on birch and PC obtained by thermal carbonization and activation with SWVA had the highest ability to capture CO_2_ and amounted to 6.43 and 6.00 mmol/g at 273 K, as well as 4.57 and 4.22 mmol/g at 298 K, respectively. CO_2_ adsorption depends on the presence of narrow micropores (<1 nm); therefore, these microporous carbons show high CO_2_ capture. Activated biocarbons were also analyzed to be effective adsorbents for the removal of Cu^2+^, Zn^2+^, Fe^2+^, Ni^2+^, Co^2+^, and Pb^2+^ ions from aqueous solutions. Activated biocarbon prepared from chemically treated CC, GS, birch, and PC with different thermal treatments appears to be an effective adsorbent for the removal of lead and zinc ions from aqueous solutions. The amount (expressed as mg·mg^–1^—adsorbate by adsorbent—) of each ion adsorbed is dependent on the pH of the metal’s solution. The optimal pH to reach the maximum adsorption capacity of the ions in the solution was in the range of 5.0–6.5. The results when analyzing the three applied models (two and three parameters), for the different activated biocarbons vs the ions adsorbed from aqueous solution, showed in general that they have a greater tendency to fit the three-parameter model (Sips) considering the parameter of *R*^2^. For the five adsorbents prepared in this investigation [CC(TC + SWVA), GS(TC + SWVA), birch (TC + SWVA), PC(TC + SWVA), and PC(HTC + SWVA)], the Cu^2+^ ion was the one with the highest adsorption capacity oscillating between 96% with the adsorbent PC(TC + SWVA) and 53% for the GS (TC + SWVA), with lower adsorption capacity. The only adsorbent in which the Cu^2+^ ion did not present the highest adsorption was CC(TC + SWVA), a material in which the Co^2+^ ions presented the highest adsorption capacity. From the above results, it can be concluded that the prepared biocarbons CC(TC + SWVA), GS(TC + SWVA), birch(TC + SWVA), PC(TC + SWVA), and PC(HTC + SWVA) can be used to remove heavy metal contamination of wastewater since they are low-cost adsorbents, and their production can be scaled to the industrial level.

## Data Availability

All data generated or analyzed during this study are included in this published article.
